# Engineering a Tumor Microenvironment‐Mimetic Niche for Tissue Regeneration with Xenogeneic Cancer Cells

**DOI:** 10.1002/advs.201700666

**Published:** 2018-01-02

**Authors:** Zhenzhen Wang, Chunming Wang, Ayipaxia Abudukeremu, Xiaying Rui, Shang Liu, Xiaoyi Zhang, Min Zhang, Junfeng Zhang, Lei Dong

**Affiliations:** ^1^ State Key Laboratory of Pharmaceutical Biotechnology School of Life Sciences Nanjing University 163 Xianlin Avenue Nanjing 210093 China; ^2^ State Key Laboratory of Quality Research in Chinese Medicine Institute of Chinese Medical Sciences University of Macau Taipa 999078 Macau SAR; ^3^ Department of Chemistry Emory University 1515 Dickey Drive Atlanta GA 30322 USA

**Keywords:** intrinsic morphology, physiological functions, regenerative medicine, tumor microenvironment‐mimetic niche, xenogeneic cancer cells

## Abstract

The insufficient number of cells suitable for transplantation is a long‐standing problem to cell‐based therapies aimed at tissue regeneration. Xenogeneic cancer cells (XCC) may be an alternative source of therapeutic cells, but their transplantation risks both immune rejection and unwanted spreading. In this study, a strategy to facilitate XCC transplantation is reported and their spreading in vivo is confined by constructing an engineering matrix that mimics the characteristics of tumor microenvironment. The data show that this matrix, a tumor homogenate‐containing hydrogel (THAG), successfully creates an immunosuppressive enclave after transplantation into immunocompetent mice. XCC of different species and tissue origins seeded into THAG survive well, integrated with the host and developed the intrinsic morphology of the native tissue, without being eliminated or spreading out of the enclave. Most strikingly, immortalized human hepatocyte cells and rat β‐cells loaded into THAG exert the physiological functions of the human liver and rat pancreas islets, respectively, in the mouse body. This study demonstrates a novel and feasible approach to harness the unique features of tumor development for tissue transplantation and regenerative medicine.

## 1 Introduction

Cell transplantation aimed at tissue reconstruction and regeneration holds promise to solve the fundamental challenges with organ transplantation, including an extreme shortage of whole organs suitable for medical implantation as well as its associated medical, social, and ethical issues.^1^, ^2^, ^3^ Ideally, the therapeutic cells are collected from the patient's own tissue to avoid immunogenic rejection, expanded ex vivo and delivered back to the patient with or without the use of scaffolds (“autograft”).^4^, ^5^ However, the number of obtainable cells is usually far insufficient to construct a functional tissue or organ.^3^ One microliter of human tissue typically contains about 10^9^ cells; and the liver, as an example of a whole organ, comprises over 10^11^ hepatocytes.^6^ It is unlikely to obtain extra billions of primary cells through isolation and ex vivo expansion because many somatic cells proliferate slowly or do not proliferate at all. This insufficiency is a long‐existing bottleneck hampering the clinical applications of cell transplantation. Besides, although theoretically stem cells have unlimited self‐renewal capacity and may provide more cells after expansion,^7^ their differentiation into desirable lineage and generation of functional tissues are hard to control.^8^ As such, cells that can rapidly proliferate and readily constitute a new tissue remain highly demanded in regenerative medicine.

Cancer cells are not normally linked with tissue regeneration, but they indeed fulfil the above requirements for transplanted cells. First, cancer cells grow fast, proliferating 3–5 folds faster than normal cell lines.^9^, ^10^ Second, many cancer cells can preserve the core functions of their tissue of origin. In tissue engineering research, cancer cells are commonly employed to test the performance of biomaterials scaffolds in vitro,^11^ as their phenotype can simulate that of the target tissue. In some artificial livers (a type of ex vivo device), hepatoma (liver cancer cells) were even used to substitute normal hepatocytes and functioned well.^12^ Third, a solid tumor has a complete structure of the organ where it arises, comprising parenchymal (neoplasm) and stromal cells, rich extracellular matrix (ECM) components, independent circulation and an immunosuppressive microenvironment.^13^, ^14^ These features of tumor would precisely favor the regeneration of many tissue types. But certainly, if cancer cells were serving as therapeutic cells, their localization is important because their migration out of the implanted sites may risk tumorigenesis in other healthy organs and must be strictly prohibited.

Nevertheless, if we transplant cancer cells for regenerative purposes, how can we promote their growth locally while preventing their migration globally at the same time? We proposed to harness the power of immunity to realize this goal. Specifically, we aimed to use xenogeneic cancer cells (XCC) and create an immunosuppressive microenvironment (an “enclave”) for these trans‐species cells to grow. However, their migration out of this niche would be naturally inhibited by the robust cross‐species immune response. First, it is common to create tumor models in cancer research by transplanting xenogeneic (human) cancer cells into immunocompromised mice—but not immunocompetent mice, where promptly triggered immunogenic rejection and could immediately eliminate the transplanted cells.^15^ Likewise, this mechanism could be utilized to confine the implanted XCC in the engineered immunogenic enclave. Second, this enclave should mimic the characteristics of tumor microenvironment (TME). Like transplanted cells, the tumor is also recognized as “foreign” by the immune system but can effectively escape immunosurveillance to flourish.^16^, ^17^, ^18^, ^19^ The key reason is the formation of its unique microenvironment, in which multiple biochemical and physical signals dynamically coordinate to educate immunocytes, including macrophages, dendritic cells (DCs) and lymphocytes—which continue infiltrating into the tumor as the blood vessels invade—into an immunosuppressive phenotype.^20^, ^21^, ^22^, ^23^ The educated immune cells, in turn, help the tumor escape immune attack, promote neoplasm growth, encourage angiogenesis, and thereby shape TME into a better platform to support cancer cell growth.^24^, ^25^ Therefore, cultivating and transplanting XCC in an engineered, TME‐mimetic matrix niche would exert the maximum potency of cancer cells for tissue regeneration while preventing their spreading in the host body.

**Scheme 1:**
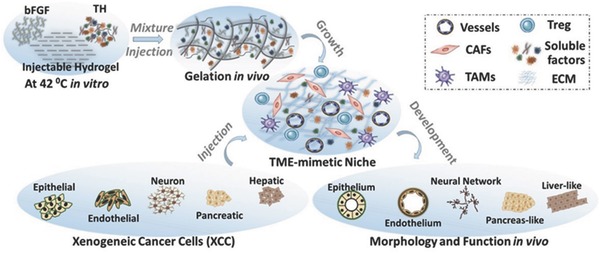
Schematic diagram of the concept that an engineered TME‐mimetic niche formed by active soluble factors in tumor extract (TH), basic fibroblast growth factor (bFGF), and injectable hydrogel facilitates the xenogeneic cancer cells (XCC) to develop into a functional tissue in immunocompetent mice.

To prove this concept, in the present study, shown as **Scheme** **1**, we aimed to design a TME‐mimicking matrix and evaluated its capability in supporting the growth of XCC in immunocompetent mice. The matrix comprised the soluble extract of a murine sarcoma (which are supposed to preserve the biological factors for TME formation), a synthesized hydrogel (which offers mechanical support and injectability) as well as recombinant proteins of basic fibroblast growth factor (bFGF) aimed at enhancing angiogenesis.^26^ The prepared gel, carrying different types of XCC, was injected subcutaneously into mice to create the immunogenic enclave. The confinement of XCC growth within the enclave and meanwhile the development of various types of xenograft tissue were evaluated. Our results demonstrated the successful formation of a TME‐mimetic niche in the hydrogel implants that could support trans‐species cell growth into specific tissue structures in the back of immunocompetent mice, with no signs of cell migration out of the created enclave. Most encouragingly, two xenograft models, originating from human hepatic‐transformed cell line (THELE‐3) and rat insulin‐producing cell lines (INS‐1), exerted normal functions of the liver and pancreas islets, respectively, in the mice body.

## 2 Results

### 2.1 Tumor Homogenate (TH) Creates a TME‐Mimetic Niche

Our goal was to fabricate a TME‐mimetic matrix, made of soluble TH and an injectable hydrogel, for the culture of xenogeneic cells. We assumed that TH contained the essential ingredients to modulate the stromal cells into a pro‐tumor phenotype which would favor the growth of transplanted cells and creation of TME‐like microenvironment. To validate this assumption, we obtained TH from the soluble extract of murine sarcoma (derived by the transplantation of S180 cell line), profiled its protein composition by liquid chromatography mass spectrometry (LC‐MS) (Data File 1, Figure S1, Table S1, Supporting Information) and tested its effects on the behavior of primary fibroblasts and macrophages. We chose these two cells for testing because cancer‐associated fibroblasts (CAF) and tumor‐associated macrophages (TAM) are the two major stromal cell populations in TME that promote tumor development.^27^, ^28^

**Figure 1:**
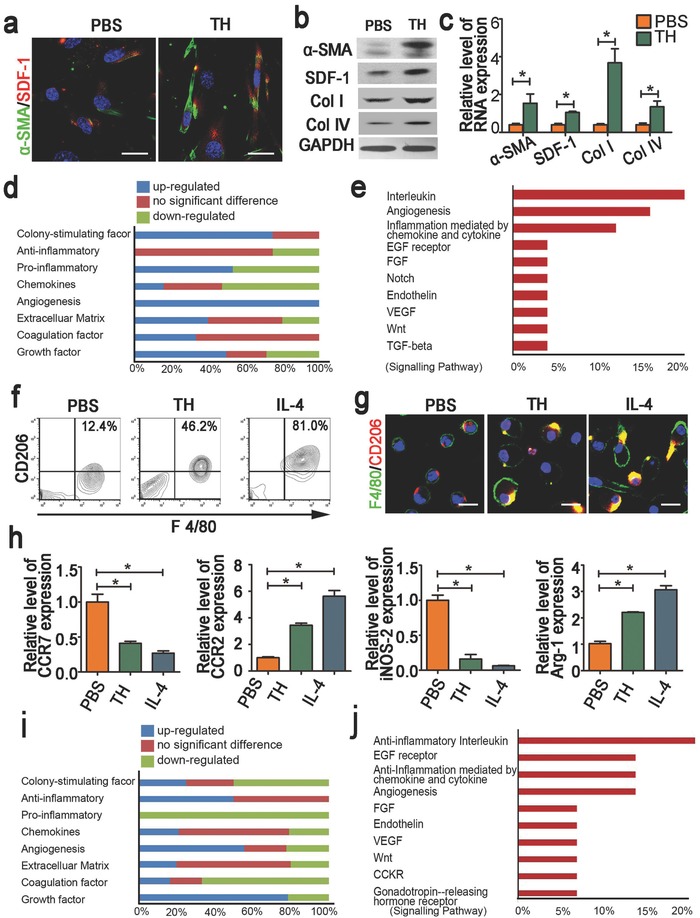
Effects of tumor homogenate (TH) on the phenotype of primary fibroblasts and macrophages. a) Representative immunostaining images illustrating the expression of α‐SMA and SDF‐1 in primary fibroblasts treated with TH or PBS (Scale bar = 50 µm); b) Western blotting, and c) RT‐PCR analysis of the expression of fibroblast makers—α‐SMA, SDF‐1 and collagen type I and IV in primary fibroblasts treated with TH or PBS; d) Hierarchical cluster analysis of 78 kinds of soluble proteins in the culture medium of primary fibroblasts treated with TH relative to that with PBS according to molecular functions; e) Pathways enriched by the up‐regulated proteins shown in the panel d with Gene Ontology Pathway analysis; f) FACS analysis and g. immunofluorescent staining of CD206 and F4/80 in primary macrophages treated with TH or PBS, with IL‐4 as positive control (Scale bar = 50 µm); h) RT‐PCR analysis of the expression of macrophage makers—CCR7, CCR2, iNOS‐2, and Arg‐1 in primary macrophages cells treated with TH, PBS, or IL‐4; i) Hierarchical cluster analysis of 78 kinds of soluble proteins in the culture medium of primary macrophages treated with TH relative to that with PBS according to molecular function; j) Pathways enriched by the up‐regulated proteins shown in the panel (i) with Gene Ontology Pathway analysis. Images are representative of three independent experiments. Results are shown as mean ± SD. *P < 0.05 after ANOVA with Dunnett's tests.

The results from immunofluorescent (IF) staining (**Figure** **1**a), Western blotting (WB, Figure 1b) and quantitative polymerase chain reaction (PCR) (Figure 1c) consistently suggested that treatment with TH for 48 h significantly stimulated the expression of α‐SMA and SDF‐1—two representative markers for CAFs—in primary mammary fibroblasts.^29^ The treatment also upregulated the levels of Type I and IV collagen, which are also highly expressed in CAF (Figure 1b and c). Notably, an antibody assay examining 78 proteins (Table S2, Supporting Information) revealed that the TH‐treated fibroblasts expressed abundant pro‐angiogenic and pro‐growth factors, which are usually secreted by CAF to promote the shaping of TME. The expression of angiogenesis‐related cytokines (100%), colony‐stimulating factors (CSF, 75%), and growth factors (50%) were highly up‐regulated (Figure 1d). Further analysis of these data with gene ontology revealed the high enrichment of pathways associated with angiogenesis and anti‐inflammation, as well as the involvement of signals relating to several growth factors (Figure 1e). These data indicated that TH could induce fibroblasts to acquire the phenotype of CAF.

We next analyzed the influence of TH on the primary bone marrow derived macrophages (BMDM). First, both flow cytometry analysis (Figure 1f) and IF staining (Figure 1g) indicated that the TH treatment up‐regulated the expression of CD206 in the macrophage population. Meanwhile, quantitative PCR analysis revealed increased levels of CCR2 and Arg‐1 and decreased expression of CCR7 and iNOS‐2 in the TH‐treated BMDM (Figure 1h). As CCR7 and iNOS‐2 are M1 markers, while CD206, CCR2, and Arg‐1 are typical M2 markers,^30^ the data suggested that TH triggered a M2‐way polarization of BMDM. Next, as revealed by the antibody assay and follow‐up ontology analysis, TH treatment upregulated the levels of CSF (25%), anti‐inflammatory cytokines (50%), pro‐angiogenic factors (60%) and growth factors (78.57%) in BMDM (Figure 1i), enriching the pathways associated with anti‐inflammation, angiogenesis and EGF receptor (Figure 1j). Thus, as it endowed the primary fibroblasts with the phenotypes and functions of CAF, TH could also transform the primary macrophages into an M2 phenotype functionally similar to TAM. These TH‐educated cells switched to secrete cytokines that were typically produced by CAF and TAM in shaping up TME. These findings validated that TH could be used in engineering scaffolds to create a TME‐mimetic niche for cancer cell growth.

### 2.2 Creation of TME‐Mimicking Microenvironment In Vivo by Implantation of THAG—A TH‐Containing Hydrogel

Having validated the effect of TH on remodeling stromal cells, we speculated whether the TME‐like niche in vivo could be constructed by physically mixing TH with an injectable hydrogel and subcutaneously implanting the mixture into mice. We prepared the hydrogel by chemically crosslinking agarose and gelatin (ACG), according to previously reported methods,^31^ and characterized it with scanning electron microscope (SEM) and IR spectrum (Figure S2a and b, Supporting Information). It also had a tunable phase transition temperature, mechanical strength, and flexibility (Figure S2c–e, Supporting Information). Then, TH (≈2–3 mg protein) was mixed with the liquid ACG (1% in PBS) at 42 °C, and the mixture solidified and became TH‐containing ACG (THAG) when the temperature decreased to 37 °C (Figure S2d, Supporting Information). THAG demonstrated excellent support of cell growth, as both fibroblasts and macrophages adhered well to its surface and proliferated both on its surface and inside its matrix (Figure S2f–h, Supporting Information). Additionally, fibroblasts and macrophages encapsulated in THAG expressed the markers of CAF (α‐SMA^high^/SDF‐1^high^) and M2‐polarization (CD206), respectively (Figure S2h, Supporting Information).

**Figure 2:**
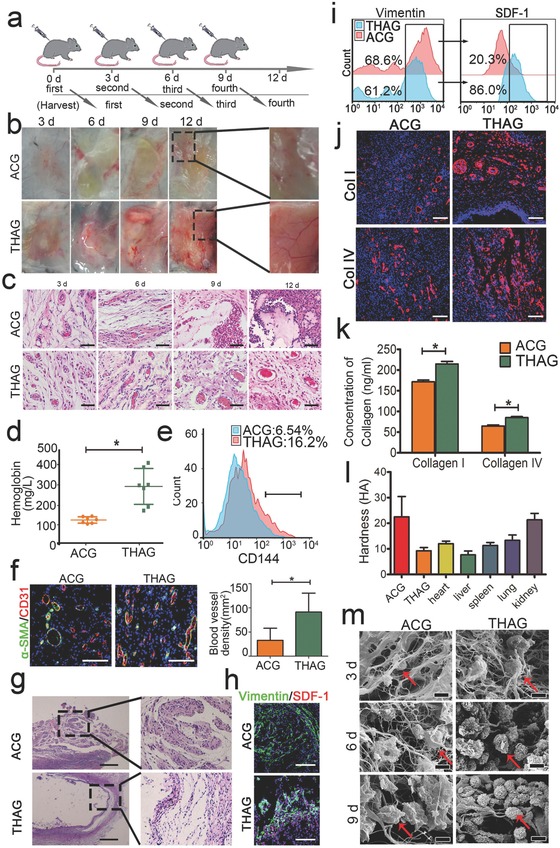
Effects of THAG, an engineered matrix mimicking tumor microenvironment, in supporting neo‐tissue formation in vivo. a) Schematic diagram of subcutaneous injection of hydrogels incorporating tumor homogenate (THAG) or saline (ACG) into the back of C57BL/6J for different times; b) Gross view of in situ neo‐tissue formation in THAG or ACG at indicated time points, with regions from Day 12 samples magnified in the right panel; c) H&E analysis of neo‐tissues in THAG or ACG implanted into the back of mice (Scale bar = 100 µm); d) Hemoglobin content in the neo‐tissue in THAG or ACG 9 days after the first injection (*n* = 7 per group); e) FACS analysis of CD144, a typical marker of endothelial cell, in neo‐tissue in THAG or ACG at Day 9; f) Representative images of immunofluorescent staining for CD31/α‐SMA in two groups at Day 9. Blood vessel density was calculated based on the confocal microscopical images shown on the right panel; g) Representative images of H&E staining illustrating foreign body reactions to THAG or ACG at Day 9(Scale bar = 250 µm); h) Representative images of immunofluorescent staining for vimentin and SDF‐1, typical fibroblast markers, in neo‐tissue in THAG or ACG (Scale bar = 100 µm); i) FACS analysis of cells expressing vimentin and SDF‐1 in neo‐tissue in THAG or ACG; j) Representative images of immunofluorescent staining for collagens type I and IV in neo‐tissue in THAG or ACG (Scale bar = 100 µm); k) Quantitative measurements of collagen contents in neo‐tissue in THAG or ACG (*n* = 7–9 per group); l) The hardness of the neo‐tissue in THAG or ACG compared with that of heart, lung, spleen, lung, and kidney (*n* = 7 per group); m) Scanning electron microscope of neo‐tissues in the two groups at indicated time points (red arrows indicate cells) (Scale bar = 20 µm). Images are representative for three independent experiments. Results are shown as mean ± SD. * *P* < 0.05 after ANOVA with Dunnett's tests.

We then injected THAG subcutaneously into the back of C57BL/6J mice every third day for four times (500 µL each time, **Figure** **2**a), with the same ACG gel with PBS as control. To further enhance angiogenesis, we added excessive bFGF (1000 U mL^−1^) in THAG (Figure S3, Supporting Information). A series of histological and cellular analyses indicated that THAG facilitated angiogenesis to a greater extent, as evidenced by a remarkably higher density of new blood vessels (gross view, Figure 2b; H&E staining, Figure 2c), more hemoglobin (Figure 2d) and CD144^+^ cells (Figure 2e), and elevated expressions of CD31 (an endothelial marker) and α‐SMA (a pericyte marker, Figure 2f). Intriguingly, the implanted THAG appeared more transparent than the ACG gels (Figure S3a, Supporting Information), indicating a mild foreign body reaction to THAG. Further histological analysis showed less foreign body granuloma formation around THAG and fewer inflammatory cells infiltrated into THAG, as compared with the ACG group (Figure 2g). On the contrary, significantly more CAFs invaded into THAG than ACG, as evidenced by IF staining (Figure 2h) and flow cytometry analysis for Vimentin and SDF‐1 (Figure 2i). Fibroblasts are adept at remodeling tissue microstructures by producing ECMs. Indeed, both Type I and IV collagens, which are crucial for the initial stage of tissue morphogenesis,^32^ were found much more abundant in THAG than in ACG (Figure 2j and k).

In addition to facilitating tissue growth, THAG also exhibited proper mechanical strength. The hardness of THAG (≈10 HA) was much comparable to that of most organs and lower than that of ACG (over 20 HA) (Figure 2l). Further SEM examination indicated that the softness of THAG was possibly owing to its degradation in vivo in response to an increase in cell density (Figure 2m).

The above data demonstrated that the implanted THAG created a desirable niche for tissue growth, with high degrees of angiogenesis and fibroblasts invasion as well as low degrees of foreign body reactions—the latter of which also suggested a successful suppression of the body's immune response against THAG and would be further investigated in the following experiments.

### 2.3 Establishment of Immunosuppressive Niche in Implanted THAG

**Figure 3:**
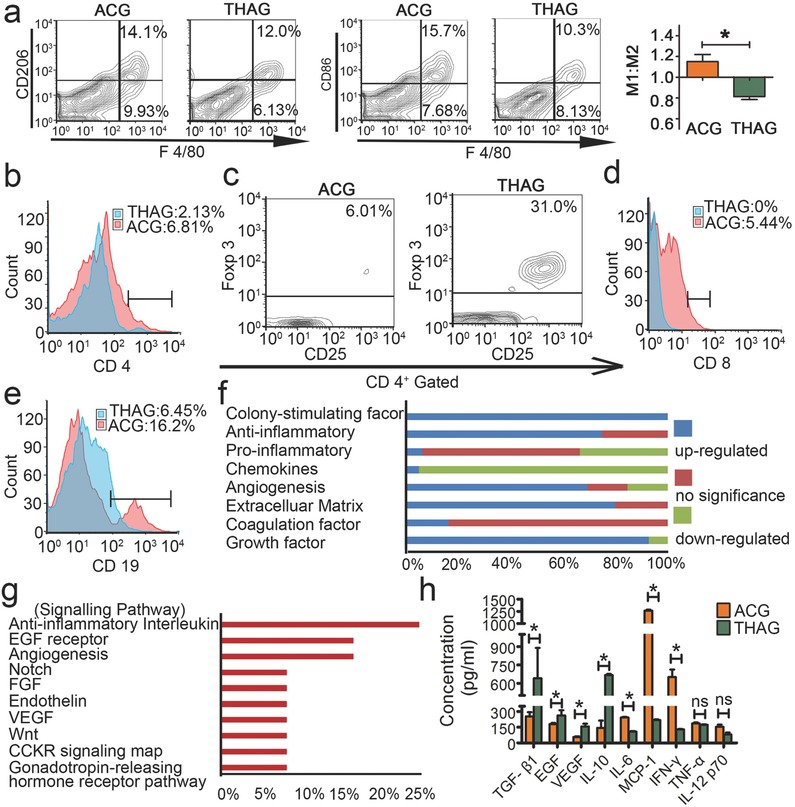
Formation of immunosuppressive environment in THAG. a) (left) Representative FACS analysis of CD206/F4/80 and CD86/F4/80 and (right) M1/M2 proportion (calculated by the number of CD86^+^/F4/80^+^ cells divided by that of CD206^+^/F4/80^+^ cells) of the cells in neo‐tissue at Day 9 after first injection of THAG or ACG; b–e). Representative FACS analysis of b. CD4^+^ T; c. CD25^+^Foxp3^+^ Treg (CD4^+^ gated); d. CD8^+^ T and e. CD19^+^ B cells in neo‐tissue as described above; f) Hierarchical cluster analysis of 78 kinds of soluble proteins in the supernatant of neo‐tissue homogenate in the animals treated with THAG relative to that with ACG according to molecular function; g) Pathways enriched by the up‐regulated proteins shown in the panel f with Gene Ontology Pathway analysis. h) The levels of growth factors (TGF‐β1, EGF and VEGF) and inflammatory cytokines (IL‐10, IL‐6, MCP‐1, IFN‐γ, TNF‐α and IL‐12p70) in neo‐tissue as in panel a. Results are shown as mean ± SD (*n* = 8–10 mice per group; *P < 0.05 versus ACG; ns = not significant).

We continued to evaluate whether THAG could create an immunosuppressive environment within its matrix after implantation in mice. This feature was crucial to our hypothesis as it served to protect the growth of XCC inside the immunologic enclave. The earlier in vitro tests showed that TH successfully educated macrophages into an immunosuppressive phenotype. Consistently, the neo‐tissue niches formed in THAG in vivo exhibited high immunosuppressive activities. First, a significantly higher proportion of macrophages (F4/80^+^) exhibited an M2‐like phenotype in THAG than in ACG, though the total numbers of macrophages were similar in these two groups (**Figure** **3**a and Figure S4, Supporting Information). Next, the number and composition of lymph cells were dramatically changed by TH—the percentage of CD4^+^ T cells was much lower in THAG than in ACG (Figure 3b). More importantly, the CD25^+^Foxp3^+^Treg cells, which play an indispensable role in maintaining immunological tolerance,^33^ was significantly higher in the CD4^+^ proportion, five times of that in ACG (Figure 3c). Besides, CD8^+^ T cells were barely detectable in THAG group (Figure 3d), where CD19^+^ B memory cells were also dramatically fewer than in ACG (Figure 3e). Further profiling of the soluble factors demonstrated up‐regulated CSF (100%), anti‐inflammatory cytokines (75%), growth factors (92.86%), angiogenesis proteins (69.23%) as well as ECM components (80%) in THAG (Figure 3f), resulting in the enriched pathways relating to anti‐inflammation, EGF signaling and angiogenesis, which are all essential for neo‐tissue formation (Figure 3g). Among the soluble factors, the levels of TGF‐beta1, IL‐10, VEGF, and EGF were determined by enzyme linked immunosorbent assay (ELISA) to be up‐regulated in THAG (Figure 3h). These cytokines are well‐known immunosuppressive and mitogenic factors.

Besides, we assessed the possible effects of TH on the normal functions of the immune system. We compared the numbers of CD4^+^/CD8^+^ T cells and CD19^+^ B cells in the peripheral blood around ACG and THAG implants against the untreated mice; we found no significant difference among these three samples (Figure S5a, Supporting Information). Consistently, we also found that the expression of IL‐10, IL‐6, MCP‐1, IFN‐γ and TNF‐α, the typical inflammatory factors, were also similar between THAG and sham‐operated control (Figure S5b, Supporting Information). These results indicated that THAG had little influence on the global immune system of the mice. As such, THAG was established as a TME‐mimetic, immunosuppressive enclave while having little influence on the global immune system, which was highly desirable according to our hypothesis.

### 2.4 Growth of XCC in Implanted THAG

**Figure 4:**
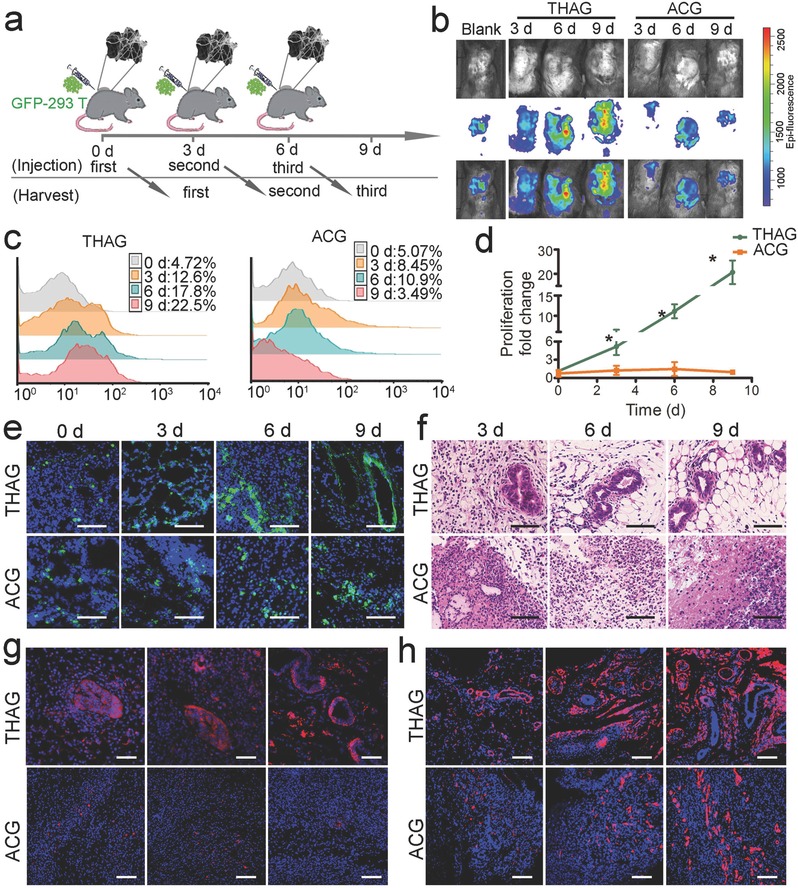
Growth of xenogeneic cell lines in THAG. a) Schematic diagram of subcutaneous injection of trans‐species 293T cells infected with lentivirus encoding GFP (GFP‐293T) into neo‐tissue in THAG or ACG. b) Bioluminescence imaging of the mice injected with GFP‐293T cells in two groups. c) FACS analysis of GFP‐positive cells in two groups treated as in panel (a). d) Quantitative analysis of GFP‐positive 293T cells calculated by multiplying the total cell number and the percentage of GFP‐293T‐positive cells (*n* = 7 per group). e) Confocal microscopical view and f) histological analysis (H&E) of GFP‐293T cells in THAG or ACG treated as in panel a (Scale bar = 100 µm); g) Images of immunofluorescent staining for human histone and h. type I collagen in THAG or ACG group treated as in panel (a) (Scale bar = 100 µm). Images are representative for three independent experiments. Results are shown as mean ± SD. *P < 0.05 after ANOVA with Dunnett's tests.

We next investigated the growth of XCC in the implanted THAG. Our hypothesis was that this artificial TME‐mimetic niche could support xenograft cells to grow in immunocompetent mice. Human embryonic kidney epithelial cell 293T transfected with lentivirus labeled with GFP (GFP‐293T) were injected into THAG or ACG implanted in mice (**Figure** **4**a). Observation with a bioluminescent imaging system through a period of 9 d demonstrated that the cells continued proliferating in THAG but did not grow in ACG (Figure 4b). Further flow cytometry analysis confirmed that the cells were proliferating and reached nearly a quarter of the total population in THAG; while they failed to grow in ACG (Figure 4c). Quantification of the above data showed that the number of 293T cells had increased by 20 folds since injection (Figure 4d).

Interestingly, during the 9‐day culture in THAG, the 293T cells underwent a typical epithelial morphogenesis and developed into a regularly‐shaped tubular structure analogous to epithelial organoids (Figure 4e and f).^34^ We further stained the tissue sections with an antibody against human histone and confirmed that these epithelium‐like tissues were of human origin, i.e., derived from the implanted 293T cells (Figure 4g). Another notable result was the abundant production of type I collagen in THAG (Figure 4h); this ECM component is required for both the growth of the implanted cells and tissue morphogenesis.

Meanwhile, we examined whether the introduction of human‐origin 293T cells would change the immunosuppressive microenvironment already established within THAG. We determined the levels of a series of inflammatory cytokines both (i) inside the THAG/ACG (Figure S6a, Supporting Information) and (ii) in the serum and different organs (Figure S6b, Supporting Information) with ELISA. The outcomes suggested that, in agreement with the earlier findings, the immune response within the THAG implants was kept low in ACG, while the global immune system was unaffected by either implants.

We also found that the implanted 293T cells did not migrate out of THAG and spread to any normal tissue in the mice body (Figure S6c, Supporting Information), which was crucial for the safe use of this method. These data suggested that THAG could provide a desirable environment for trans‐species tissue growth, by not only supporting the proliferation of xenograft cancer cells but also facilitating the reconstitution of their native morphologies. These advantages may favor the development of xenograft cells into specific tissue structures in vivo.

### 2.5 Formation of Specific Tissue Structures by Xenograft Cells in Implanted THAG

**Figure 5:**
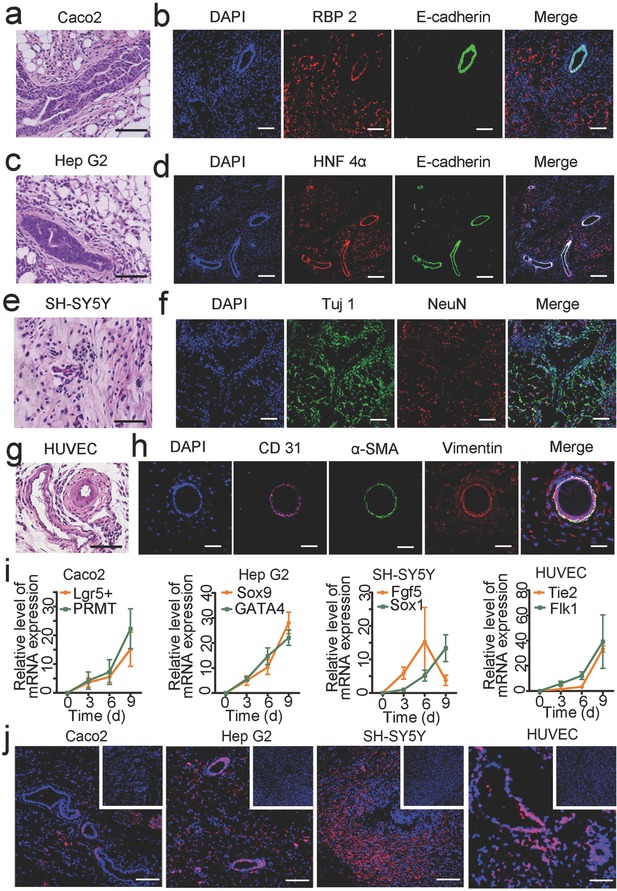
Morphology of specific tissue structures formed by human cell lines in THAG. a) Histological examination (H&E staining) of epithelioid structures and b) immunofluorescent staining for retinol‐binding protein II (RBP 2, marker of Caco2 cells) and E‐cadherin (marker of epithelium) in Caco2 cells‐laden THAG implanted in mice; c) Histological examination (H&E staining) of epithelioid structure and d) immunofluorescent staining for hepatic nuclear factor 4 alpha (HNF 4 α, marker of Hep G2 cells) and E‐cadherin in Hep G2 cells‐laden THAG implanted in mice; e) Histological examination (H&E staining) of neonatal neuron tissue and f) immunofluorescent staining for Tuj 1 and NeuN (both markers of neuron cells) in human neuroblastoma epithelial cell SH‐SY5Y‐laden THAG implanted in mice; g) Histological examination (H&E staining) of vascular structure and h) immunofluorescent staining for human CD31 (marker of endothelium), mouse α‐SMA and vimentin (markers of tunica media and external) in primary human umbilical vein endothelial cell (HUVEC)‐laden THAG implanted in mice; i) RT‐PCR analysis of specific markers of the epithelial, neural and vascular structures (*n* = 7–9 per group); j) Immunofluorescent staining for human histone expressed by different xenograft cells in THAG (those in ACG as control shown in the inset figures). Images are representative for three independent experiments. Scale bar = 100 µm. Results are shown as mean ± SD.

The above findings that 293T cells could undergo epithelial morphogenesis in THAG inspired us to ask whether other types of xenograft cells would do so when implanted into THAG. We selected four representative cell lines—including two epithelial cells Caco2 and Hep G2, one neural cell line SH‐SY5Y and one endothelial cell line HUVEC—and implanted them into THAG or ACG in the back of immunocompetent mice. All of them grew normally in THAG but not in ACG (Figure S7, Supporting Information). Remarkable epithelial morphogenesis was recapitulated in the neo‐tissues formed in the cell‐laden THAG, where Caco2 and Hep G2 cells developed into tubal organoids (**Figure** **5**a,c, Figures S8 and S9, Supporting Information).^35^, ^36^ IF co‐staining for human retinol‐binding protein II (RBP 2) or human hepatic nuclear factor 4 alpha (HNF 4α)—markers of Caco2 and Hep G2 cells,^34^, ^37^ respectively—and E‐cadherin further testified that the tubular structures were constituted by the formed epithelium organoids (Figure 5b and d).

In the human neuroblastoma (epithelial cell SH‐SY5Y)‐laden THAG, the neonatal neuron tissue successfully developed during the period of the experiment (Figure 5e and Figure S10, Supporting Information). At day 3 after the first injection, neuroepithelial cysts, one of the early stage structure of neuroepithelium, developed in the THAG group and significantly expressed E‐cadherin (Figure S11a, Supporting Information).^38^ At day 8, which was 5 d after the second injection, neurons started to grow basally. Increasing numbers of cysts were observed, with up‐regulated N‐cadherin and down‐regulated E‐cadherin (Figure S11b, Supporting Information). At day 12, or 6 days after the third injection, the cells with cysts continued neural differentiation by extending dendrites and forming network, as evidenced by the staining for neurogenesis markers NeuN and βIII‐tubulin (Tuj 1) (Figure 5f).

In addition to these cancer cells, HUVEC, which are normal primary endothelial cells of human origin, also formed tubular structures after implantation into THAG (Figure S12, Supporting Information and Figure 5g). Moreover, the implanted human cells successfully recruited tunica external and media cells from the mouse body to form a mature vasculature structure,^39^ as evidenced by IF staining for human CD31 (endothelial layer, internal), anti‐mouse α‐SMA (smooth muscle cells, middle), and anti‐mouse vimentin (connective tissue, external; Figure 5h). Besides, the transcriptional levels of both Lgr5+/PRMT and Sox9/GATA4, which are important genes during intestinal and hepatic epithelium generation,^40^, ^41^ were up‐regulated as Caco2 and Hep G2 grew. Similarly, early neurogenic markers, such as Fgf5 for primitive ectoderm and Sox1 for neuroectoderm,^38^ were expressed along the cultivation of SH‐SY5Y in THAG. And the levels of Flk1 and Tie2, two markers of endothelial development,^39^ also increased as the humanized vessels developed in THAG (Figure 5i). Further staining for species‐specific human histone confirmed that these well‐developed tissues with morphological features were derived from the seeded human cells (Figure 5j).

In summary, these results suggested that the microenvironment in THAG not only accommodated the growth of different xenograft human cells but also supported their development into specific tissues. Remarkably, these human tissues de novo formed in the mouse body underwent normal differentiation, organized into correct structures and exhibited typical morphologies.

### 2.6 Physiological Functions of the Xenograft Tissues in THAG

**Figure 6:**
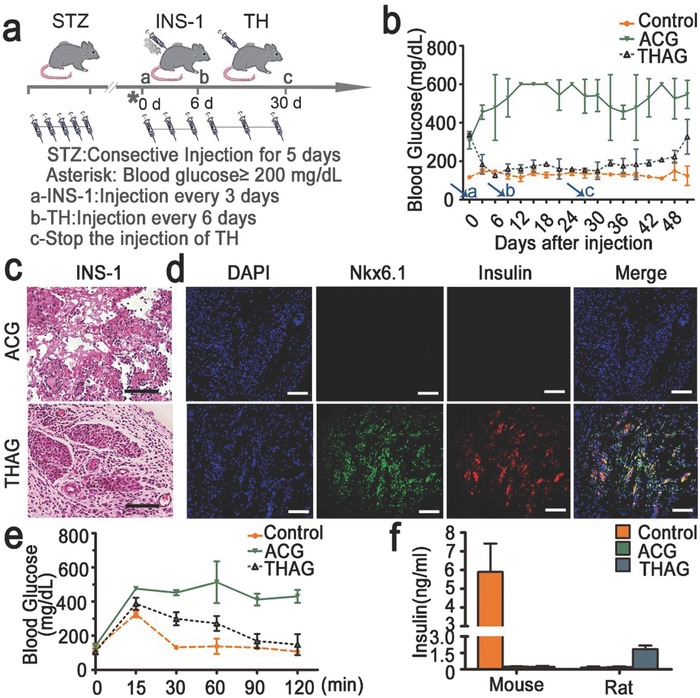
Function of INS‐1 cells in the rat ‘pancreas islet‐like’ tissue created in THAG in mice. a) Schematic diagram of diabetes reversal by subcutaneous injection of INS‐1 cells into neo‐tissue in THAG implanted in the back of mice; b) The levels of blood glucose monitored every third day following the diabetic mice were generated. The arrows named as a, b and c, respectively, indicate the corresponding treatment described as in panel (a); c) Histological examination (H&E staining) and d) immunofluorescent staining for insulin and nuclear protein Nkx6.1 of neo‐tissue 27 days after the first injection of INS‐1 into the THAG or ACG implanted in mice; e) Blood glucose levels monitored at 0, 15, 30, 60, 90 and 120 min after glucose stimulation (2 g kg^−1^) during the intraperitoneal glucose tolerance tests (IPGTT) 27 d after the injection of INS‐1 cells; f) Rat and mouse insulin in the serum of different groups measured at 30 min during IPGTT treated as in panel e. Images are representative for three independent experiments. Scale bar = 100 µm. Results are shown as mean ± SD (*n* = 7–9 per group).

To test whether the xenograft tissues could exert normal physiological functions, we implanted a rat β‐cell line (INS‐1) and an immortalized human hepatocyte cell line (THLE‐3) into THAG created on the back of type I diabetic and normal mice, respectively. We induced diabetes in THAG‐ or ACG‐implanted C57BL/6J mice by intraperitoneally injecting streptozotocin (STZ) for consecutively five days at a dose of 45 mg kg^−1^. Blood glucose levels were monitored every third day; only mice with blood glucose levels above 200 mg dL^−1^ for two consecutive days were considered diabetic and received subsequent INS‐1 cell injection (**Figure** **6**a).

Implantation of INS‐1 cells efficiently lowered the level of blood glucose in the THAG‐bearing mice after three days, and this effect maintained through a period of 27 d, during which THAG was replenished every 6 d. We intentionally stopped replenishing THAG at day 27, and the animals returned to hyperglycaemia after another 18 d. On the contrary, these cells failed to change the condition at all in the ACG‐bearing mice throughout the 45 d (Figure 6b). A set of histological examinations coupled with IF staining revealed a clear, Langerhans‐like structure expressing insulin and the nuclear protein Nkx6.1 in the new tissue within THAG (Figure 6c,d and Figure S13, Supporting Information).

Furthermore, we tested the response of this islet‐like xenograft tissue to glucose challenge—the intraperitoneal glucose tolerance test (IPGTT). The mice were fasted for overnight and intraperitoneally administrated with glucose (2 g kg^−1^ body weight) before their blood glucose levels were monitored at the indicated time points. The IPGTT outcomes indicated that the level of blood sugar was controlled well in the THAG‐bearing mice, suggesting that the “pancreas islet‐like” tissue developed in THAG secreted insulin sensitively and timely in response to glucose (Figure 6e). Interestingly, we confirmed that the insulin presented in the serum of the THAG‐bearing mice was of rat origin, i.e. produced by the xenograft cells (Figure 6f).

**Figure 7:**
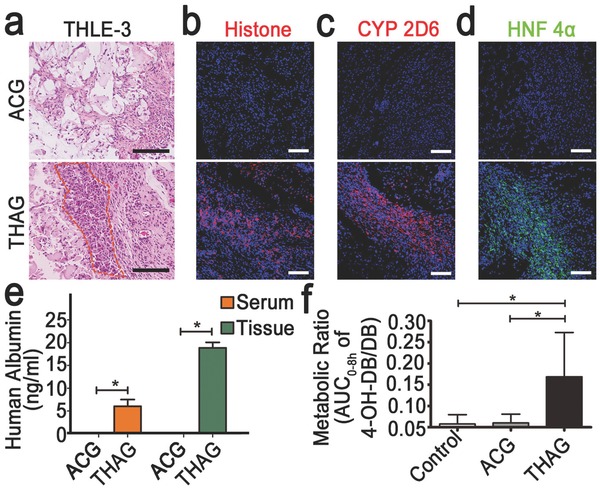
Function of THELE‐3 cells in the human “liver‐like tissue” developed in THAG in mice. a) Histological examination (H&E staining) of THLE‐3 cells‐laden THAG or ACG implanted in mice; the outlined regions highlight the large sized cells in polygonal shape, which is a typical morphology of liver cells; b) Immunofluorescent staining for human histone and c) CYP2D6 (specifically expressed by human liver cells) and d) Nuclear protein HNF 4α in the samples as in panel (a); e) The levels of human albumin detected in serum and local tissues of the mice implanted with THLE‐3 cells‐laden THAG or ACG; f) Metabolic ratios determined by dividing the AUC0‐8h (the area under the curve from time 0 until 8 h) ratio of 4‐hyroxydebrisoqune (4‐OHDB) to that of debrisoquine (DB) in untreated mice as well as mice implanted with THLE‐3 cells‐laden THAG or ACG. Images are representative for three independent experiments. Scale bar = 100 µm. Results are shown as mean ± SD (*n* = 7–9 per group). *P < 0.05 after ANOVA with Dunnett's tests.

Having validated the function of xenograft pancreas‐like tissue growing in THAG, we generated a “humanized liver‐like tissue” using the same approach and examined its function in mice. The injected human liver epithelial cells (THLE‐3) demonstrated a typical large, polygonal morphology of the liver cells (**Figure** **7**a and Figure S14, Supporting Information), expressing human histone, CYP2D6 and HNF 4α (both are markers of liver cells) in the newly formed tissue in THAG (Figure 7b–d). These humanized, liver‐like tissues faithfully exerted the function of the liver, producing human serum albumin at 6.053 ng mL^−1^ in serum and up to 18.862 ng mL^−1^ in the new tissue growing from THAG, starting at around day 10 (4 d after the third injection of THAG). However, the transplanted cells into ACG failed to produce albumin (Figure 7e). Moreover, after administrating the mice with debrisoquine (DB), which human and mice metabolized differently,^42^ we detected 4‐hyroxydebrisoqune (4OH‐DB)—a human‐specific metabolite—in the urine collected from mice implanted with THLE‐3‐laden THAG. Whereas, this agent could not be metabolized by the mice with ACG (Figure 7f). Thus, the THAG‐supported liver‐like tissue could secrete human albumin and performed drug metabolism as the human liver does. These findings suggested that THAG created in the mouse body not only supported the growth of human or rat cell lines into specific tissues, but also facilitated these trans‐species tissues to exert their typical physiological functions in the mouse body.

## 3 Discussion

One of the most exciting goals in regenerative medicine is to develop functional tissue/organs in the body using transplanted cells. To date, attempts towards this aim have been substantially hampered by several major obstacles, notably including the lack of sufficient cells suitable for transplantation and immune rejection against the “foreign” cells after transplantation.^3^, ^43^ In this study, we showed that xenogeneic cancer cells, delivered in an engineered matrix mimicking the TME, could successfully generate new functional tissues in vivo without being eliminated by the host immunity. These cells demonstrated an unparalleled (and underappreciated) potential in serving as a new, ample source of therapeutic cells for transplantation, while the TME‐mimetic niche played an indispensable role in supporting the survival and function of these cells.

A key inspiration to our investigation was the similarity between the tumor and transplanted tissues—both are recognized as “foreign” by the host immunity while both require blood supply (which inevitably brings immunocytes) from the host.^44^, ^45^ The tumor could tactically solve this dilemma thanks to its unique TME with two distinctive features—pro‐tumor growth and immunosuppressive.^17^, ^18^, ^46^, ^47^ Thus, we assumed that re‐creation of analogous TME would provide the proper ground to support xenograft cells to grow in the host body. Since little was known about the detailed mechanisms of TME formation,^48^, ^49^ the most efficient and straightforward way to establish a TME‐mimetic niche would be implanting a tumor homogenate‐containing hydrogel matrix (THAG) in vivo, as we eventually performed. Our strategy proved successful. A comprehensive set of data showed that THAG preserved the biological characteristics of TME and effectively developed into an immunoprotective, vascularized niche. Such niche, as expected, effectively facilitated the settlement of xenogeneic cells of various tissue origins—from human kidney, liver, intestine, endothelium, neuron to rat pancreas—and promote their development into specific, fully functional tissues in immune competent mice.

Notably, in agreement with our hypothesis, the engineered THAG not only created optimal conditions for tissue growth but also prevented immunogenic rejection from the host—which is the major cause of failure for xenografts and allografts.^15^ Although some recent studies devised “immuno‐isolating” materials to prevent the infiltration of immunocytes into the implants,^50^ such protection also cut off blood vessels and exclude the entry of other stromal cells that are essential for the neo‐tissue formation, leading to low vascularization and poor host integration that eventually cause implant failure. By creating the TME‐mimicking niche, our approach successfully translated the tumor's strategy to escape immune attack into the growth of xenogeneic tissues.

Our data showed that THAG was well integrated with the circulation system of the mice body while received minimal foreign body reactions. Instead of being barred from the implants, macrophages and fibroblasts—the two major stromal cells of high plasticity—abundantly infiltrated into THAG, vastly changed their phenotypes and actively shaped up the immunosuppressive enclave by further regulating the functions and numbers of the other stromal cells, such as the endothelial cells and lymph cells. As a result, inside the enclave, the transplanted “human” or “rat” tissues were formed, without being eliminated by the host immune rejection; out of the enclave, neither the spreading of xenogeneic cells nor the compromise in the host immunity was found. These findings collectively highlighted the efficacy and safety of THAG as an engineering matrix for tissue regeneration.

There are at least two interesting directions for furthering our exploration. First, though we have validated the effect of tumor homogenate in creating TME and analyzed its components with proteomic tools, it is also a critically deficient aspect for its possibly uncontrollable effects on the body, especially the immune system. We still need to investigate the mechanism in this process and identify the key components out of the many, to make the process more controllable. Second, though we had foreseen the growth of xenogeneic cancer/immortalized cells in THAG, we did not predict the intriguing outcomes of morphogenesis, which indicated a considerable extent of differentiation of these cell lines in THAG. Further investigations are in demand to explore the mechanisms underlying their morphogenesis. Besides, according to the properties of the engineered tissues demonstrated in our study, we prospect the technology is more suitable to construct endocrine organs, such as the pancreas, than to engineer other types of organs. In the future, it is challenging and inspiring to design more complex tissues or organs with elaborated improvement of the present method.

In summary, in the present study, we demonstrated the creation of an engineered matrix that exerts the unique features of TME to support the growth of xenogeneic tissues. The designed THAG matrix, an injectable hydrogel system incorporating tumor extract as an active ingredient, could enable the survival, proliferation and function of various xenogeneic cell lines in immunocompetent mice. Using this approach, pancreas and liver tissues respectively from rat and human cells were successfully developed in THAG implanted into the back of mice. Our findings suggest that application of a TME‐like, immunosuppressive niche, in combination with employment of xenogeneic cells, may potentially solve two fundamental challenges in cell transplantation—i.e., low cell availability and immune rejection. The novelty, feasibility, and openness of our approach may inspire the design of new strategies for tissue engineering and other cell‐based therapies.

## 4 Experimental Section

*Reagents*: Agarose, gelatin, *N*,*N*′‐Carbonyldiimidazole (CDI), dimethyl sulfoxide (DMSO), STZ, and all other chemicals used in this study were purchased from Sigma‐Aldrich (St. Louis, MO, USA) unless otherwise stated. Interleukin‐4 (IL‐4) and bFGF were purchased from PeproTech (New Jersey, USA). Debrisoquine and 4‐hyroxydebrisoqune were obtained from Toronto Research Chemicals (TRC, Toronto, Canada).

*Synthesis and Characterization of Agarose‐CDI‐Gelatin Conjugate Hydrogel*: ACG were synthesized according to a previously reported method.^31^ Briefly, agarose powder (4%; w/v) was suspended in DMSO, heated at 80 °C to dissolve and cooled down to room temperature. Next, CDI (1%) was added to activate the hydroxyl groups on the sugar chain of agarose. Subsequently, gelatin (6%) was mixed with the solution and stirred overnight at room temperature. The resulting solution was placed in a dialysis tube, dialyzed against distilled water to remove DMSO and remaining CDI and lyophilized to obtain ACG. ACG was characterized by SEM (SFEG Leo 1550, AMO GmbH, Aachen, Germany) and Fourier's transform infrared spectroscopy spectra (Shimadzu Corp., Kyoto, Japan) with the 4000–400 cm^−1^ scanning range. The mechanical properties of the hydrogel were tested using a table‐top material tester (EZ‐Test‐500 N; Shimadzu, Kyoto). Briefly, the solutions were poured into cylindrical tubes and cooled in an ice bath. The obtained cylindrical gels were compressed at a crosshead speed of 3.0 mm min^−1^ for the stress–strain profiles test. Rheological measurements were performed in a TA Instruments AR1000 Rheometer using the parallel plate shear mode. Dynamic viscoelastic measurements were performed to measure the storage modulus, G′, the loss modulus, G″, and the loss angle tangent, tan (delta). ACG hydrogel solutions of different concentration were examined with the temperature sweeps between 65 and 25 °C.

*Extracts from TH and Proteomics Analysis for LC‐MS Profile of TH*: To generate the heterotopic tumor model, mouse sarcoma cell line S180 cells (1 × 10^6^) were injected subcutaneously into the left arm pits of the animals. Mice bearing implanted tumors were sacrificed when the sizes of the implanted tumors reached about 0.5 cm. The tumors were removed, immersed in ice‐cold phosphate buffered saline pH 7.4 (PBS), minced, and washed with the same solution. The mince was homogenized with a Teflon/glass homogenizer. The homogenate was centrifuged at 12 000 rpm for 10 min at 4 °C. The pellets were discarded and the supernatant was collected as TH. The protein in the supernatant was quantified using Bradford assay (Biorad, CA, USA) and then stored at −80 °C. The composition of TH was analyzed by label‐free LC‐MS adhered to a method described previously.^51^ Briefly, total protein (100 µg) was reduced by adding dl‐dithiothreitol (Sigma‐Aldrich) (1 m, 60 °C, 1 h), and free cysteines were alkylated with 1 m iodoacetamide (Sigma‐Aldrich) (room temperature, 10 min in the dark). The alkylated proteins were further washed with 100 × 10^−3^ m tetratehylammonium bromide for three times at 4 °C for 20 min by centrifugation at 12000 rpm. Then the protein was digested with porcine sequencing grade trypsin (LC‐MS Grade, Sigma‐Aldrich) overnight at 37 °C. The samples were then subjected to LC‐MS analysis on a Shimadzu UFLC 20ADXR HPLC system in‐line with an AB Sciex 5600 Triple TOF mass spectrometer (AB SCIEX, Framingham, Massachusetts State, USA). Samples were analyzed in three technical replicates. Identification of peptides and proteins from continuum LC‐MS data was performed with the ProteinPilot 4.5 software (AB SCIEX), based on the Paragon database search algorithm. Proteins were analyzed by searching the mouse taxon of the UniProtKB/ SwissProt database (release 2011_11) using the precursor and fragmentation data provided by the LC‐MS acquisition method. Then, LC‐MS profile of TH was analyzed by Gene Ontology (http://geneontology.org/) according to biological process, molecular function, and cellular component or with gene ontology pathway database. Both “GO analysis” and “Pathway analysis” were analyzed in the standard enrichment computation method.

*Cells Preparation, Isolation, and Treatment—Cell Preparation*: Human embryo kidney epithelial cell 293T, human colon epithelial cells Caco2, human liver epithelial cells Hep G2, human neuroblastoma cells SH‐SY5Y, primary human umbilical vein endothelial cells (HUVEC), rat insulinoma beta cell INS‐1, and immortalized human liver epithelial cells THLE‐3 were obtained by Stem Cell Bank, Chinese Academy of Sciences (Shanghai, China). GFP stably expressing 293T cells (GFP‐293T) were sorted in the presence of puromycin (10 µg mL^−1^; Sigma‐Aldrich) after the cells were transfected with GFP‐labeled lentivirus carrying puromycin‐resistant marker. Cells were cultured in DMEM or RPMI 1640 medium containing 10% fetal bovine serum (Thermo Scientific, MA, USA), harvested at ≈80% confluence, washed twice with phosphate buffer saline (PBS), counted and re‐suspended in PBS before injection.

*Isolation and Treatment of Primary Mammary Fibroblast*: The primary fibroblasts were isolated form mammary glands of female C57BL/6J mice according to a reported protocol.^52^ Briefly, glands were digested with collagenase I and hyaluronidase in DMEM/F12 (Thermo Scientific). After digestion, the tissues were washed and cultured in DMEM/F12 media supplemented with 10% FBS and 1% penicillin/streptomycin at 37 °C. The cells were sub‐cultured after the cultures reached confluency. After 2–3 passages, stromal fibroblasts in high homogeneity were obtained. All the stromal fibroblasts used in the experiments were at less than ten passages to maintain the closest phenotype to the primary tissues. To analyze the effects of TH on the primary fibroblast, the cells were treated with TH (30 µg mL^−1^) for 48 h.

*Isolation and Treatment of Bone Marrow Derived Macrophages*: BMDM were collected and cultured according to a published protocol.^53^ Briefly, bone marrow cells from the femurs of C57BL/6J mice were harvested by flushing with Hanks' balanced salt solution without Ca^2+^/Mg^2+^. A single‐cell suspension was created by passing through a 21‐gauge needle. Non‐adherent cells were removed after culture for 4 h and purified monocytes were incubated for 7 d in RPMI 1640 supplemented with FBS (10%) and M‐CSF (50 ng mL^−1^) to obtain macrophages. Activation of BMDM was carried out by the addition of TH (30 µg mL^−1^) for 48 h. M2 macrophages induced by IL‐4 (20 ng mL^−1^, overnight) were set as positive control.

*Cell Growth on or in TH‐Incorporated ACG In Vitro*: The gel plates were prepared by pouring of 1% ACG (2.0 mL) or THAG (ACG mixed with TH) into each well of the 6‐well culture plates. Primary fibroblast or macrophage cells were seeded on the gel plates (5 × 10^5^ cells/well) and cultured for 1, 3, and 5 d. CCK‐8 kit (Dojindo Laboratories, Kumamoto, Japan) was used to examine the proliferation of these two cells at indicated time points, with cells cultured in monolayer on tissue culture polystyrenes (TCPS) as control. After 3 d, the live cells were stained with Calcein‐AM (Thermo Scientific) and their morphology was assessed under a TE2000‐U inverted phase‐contrast microscope (Nikon, Tokyo, Japan).

Furthermore, to analyze the morphology of these cells in THAG in vitro, primary fibroblast cells or macrophage cells were mixed into the pre‐sterilized THAG (1%) and deposited into each well to reach a final cell density of 5 × 10^6^ cells mL^−1^. The mixture was incubated at 37 °C for gelation. Then, cell culture medium was added and was changed every other day.

*Xenograft Cell Implantation Model*: Male or female C57BL/6J mice (20 ± 2 g) of the same ground were obtained from Model Animal Research Centre of Nanjing University (Nanjing, China). All animals had free access to rodent chow and water, and were treated in strict accordance with the institutional ethical regulation on animal experiments. Animal protocols were reviewed and approved by the Animal Care and Use Committee of Nanjing University, and conformed to the Guidelines for the Care and Use of Laboratory Animals published by the National Institutes of Health.

*In Vivo Gelation Model*: To generate the gelation model in vivo, the hydrogel solution was subcutaneously injected into the back of mice at 42 °C, and the injection site was immediately cooled by ice compress to quickly solidify the gel, based on the thermo‐reversal of 1% ACG from liquid form at 42 °C to solidified state at 37 °C (or lower temperature). ACG or ACG incorporated with TH (≈2–3 mg proteins; THAG) in sterile physiological saline mixed with or without bFGF (1000 U mL^−1^) in total 500 µL volume was injected into C57BL/6J mice continuously for different times every third day. At the indicated time points, the neo‐tissue niches formed in the injection site were extracted and subjected to histological analysis.

*Xenograft Cell Implantation*: After the gel formation in vivo, different kinds of xenograft cell lines (5 × 10^7^; GFP‐293T, Caco2, Hep G2, SH‐SY5Y, HUVEC and THLE‐3) mixed into 1% ACG or THAG (both incorporating bFGF) were injected into the site of gels for three times.

*Intravital Imaging*: Mice implanted with xenograft GFP‐293T cells were monitored by intravital imaging. Briefly, 3 d after each injection, the animals were anesthetized with isoflurane and the hair at the site of injection was removed. GFP bioluminescence in the dorsum of mice was imaged by IVIS Lumina XR system (PerkinElmer, Waltham, MA, USA) at an excitation wavelength of 488 nm. The obtained images were analyzed using Velocity 3D Image Analysis Software (PerkinElmer, Waltham, MA).

*Determination of Proteins by Enzyme Linked Immunosorbent Assay*: Serum or supernatant of tissue homogenate was collected and frozen at −80 °C before use. The levels of human albumin (ALB), rat insulin, mouse insulin, collagen type I, collagen IV, growth factors (TGF‐β 1, EGF, VEGF), and typical inflammatory factors (IL‐6, IL‐10, MCP‐1, IFN‐γ, TNF‐α, and IL‐12 p70) were measured using corresponding ELISA Quantitation Kits (Abcam, UK) according to the manufacturer's instructions.

*Profiling of Soluble Factors by Mouse Cytokine Array Kit and Mouse Angiogenesis Array Kit*: To investigate the effect of TH on the tissue‐forming niches and the functional change of fibroblasts and macrophages, the supernatant of tissue homogenate and these two kinds of cells treated with TH were collected. Their soluble components were analyzed by membrane‐based antibody assay kit for 78 kinds of different soluble factors (R&D, USA; including Proteome Profiler Mouse Cytokine Array kit and Mouse Angiogenesis Array Kit) according to the manufacturers' protocols. The expression profiles of these 78 factors underwent hierarchical cluster analysis for molecular functions by Gene Ontology. The up‐regulated proteins were further investigated with Gene Ontology Pathway database, with pathways ranking within top 10 listed.

*Quantitative Hemoglobin Assay*: The hemoglobin in tissue was detected with a quantitative hemoglobin assay kit (Nanjing Jiancheng Bioengineering Institute, Nanjing, China) according to the manufacturers' instructions. Briefly, the supernatant of neo‐tissue niches homogenate was mixed with potassium ferricyanide and potassium cyanide for 5 min. Absorbance was determined at 540 nm on a microplate reader. The results were referenced to a standard curve made by cyanmethemoglobin in different concentrations.

*The Hardness Test*: The hardness of neo‐tissue niches and other tissues were measured using an elasticity‐measuring instrument equipped with a coil spring (Type A Durometer) (CL‐150SL, ASKER, Japan). A constant force was applied to each tissue sample and the mean value representing three individual tests was recorded.

*Flow Cytometry Analysis*: Cells or tissues were digested to generate a single‐cell suspension, which was then blocked with 1% bovine serum albumin and incubated with the fluorescence‐conjugated monoclonal antibodies specific for the following cell surface markers in the dark for 30 min at 4 °C: CD206, CD86, F4/80, CD4, CD8, CD19, CD25, Foxp 3, CD144, vimentin, and SDF‐1 (eBioscience, MA, USA). The samples were centrifuged at 400–500 × *g* for 5 min at 4 °C to remove unbound antibody. After rinsing for three times, each sample was resuspended for analysis using a BD fluorescence activated cell sorter (FACS) Calibur (BD Biosciences, San Jose, CA, USA). Unconjugated antibodies and IgG controls were run in parallel to set the background. Besides, to analyze the growth of GFP‐293T cells as xenografts, the neo‐tissue was digested and subjected to FACS. The number of GFP‐positive cells was calculated by multiplying the total cell number in the injection site and the percentage of positive cells.

*Western Blotting*: Proteins were separated by SDS‐PAGE and transferred onto the polyvinylidine difluoride membranes. The membranes were blocked with skim milk and incubated with diluted primary antibody—SDF‐1, α‐SMA, collagen type I (Col I), collagen type IV (Col IV), and glyceraldehyde‐3‐phosphate dehydrogenase (Abcam, Cambridge, MA) at 4 °C with gentle shaking overnight. After five times of washing with PBST (PBS with 0. 1% Tween‐20), the membranes were probed with horseradish peroxidase‐conjugated anti‐rabbit, anti‐mouse, or anti‐goat IgG (Life Technologies, Grand Island, NY, USA) for 1 h at room temperature. After rinsing, bands were visualized with fluorography using an enhanced chemiluminescence system (Cell Signaling Technology).

*RNA Isolation and Quantitative Real‐Time PCR*: Total RNA from cells or tissues was extracted by using Trizol (Life Technologies). Real‐time PCR was performed by using a SYBR Prime Script RT‐PCR Kit (Takara Bio, Shiga, Japan) in an ABI 7300 Fast Real‐time PCR System (Applied Biosystems, FosterCity, CA). Each sample was analyzed in triplicates and repeated for three or four independent assays. The level of each gene was normalized to that of β‐actin. Primers used are listed below (Invitrogen, Carlsbad, CA, USA):

**Table:** 

Name	Forward (5′‐3′)	Reverse (5′‐3′)
α‐SMA	GAGCGTGAGATTGTCCGTGA	GGTGCTGGGTGCGAGG
SDF‐1	CTCTGCATCAGTGACGGTAA	CTCTTCTTCTGTCGCTTCTT
Col IV α1	TATGTCCAAGGCAACGAGC	AACCGCACACCTGCTAATG
Col I α	CAACAGTCGCTTCACCTACAGC	GTGGAGGGAGTTTACACGAAGC
CCR2	CTCTACATTCACTCCTTCCACT	TACAAACTGCTCCCTCCTT
CCR7	TTCAACATCACCAATAGCAG	GAAGGCATACAAGAAAGGG
Arg‐1	AACACGGCAGTGGCTTTAACC	GGTTTTCATGTGGCGCATTC
iNos‐2	CAGCTGGGCTGTACAAACCTT	CATTGGAAGTGAAGCGTTTCG
GATA4	TGGCGTCTTAGATTTATTCAGGTTC	TGTGCCAACTGCCAGACTACC
PRMT	GGAACACTCAATCCCAATAACC	CTACTTTGACTCCTATGCCCACT
Lgr5+	GTCAGTGTTCTTAGTTCAGGCAAAT	CGTTCGTAGGCAACCCTTCTC
Sox4	CTTTTCCCCTTTCTCCTTCTA	TCTAACCTGGTCTTCACCTACTG
Fgf5	TCTCCTTTTATCTGCCCCCT	GAGCAGATGCACTCATTCCA
Sox1	CGAGCCCTTCTCACTTGTT	TTGATGTTGGGGGTAT
Sox9	AGGAAGCTGGCAGACCAGTA	TCCACGAAGGGTCTCTTCTC
Flk1	GGCTCTTTCGCTTACTGTTCT	CCTGCCTACCTCACCTGTTTC
Tie2	AGGGAGTCCGATAGACGCTGT	GGACCCATCAAATCCAAGAAG
β‐actin	GGTGTGATGGTGGGAATGGG	ACGGTTGGCCTTAGGGTTCAG

*The Diabetic Mice Model*: The insulin‐dependent diabetic mice were prepared after the formation of neo‐tissues by intraperitoneal injection of STZ (45 mg kg^−1^; in acetate phosphate buffer, pH 4.5) to C57BL/6J male mice for consecutive 5 days. The mice were considered diabetic when their blood glucose levels exceeded 200 mg dL^−1^ for two consecutive days.

*The Pancreas Islet‐Like Xenograft Tissue Model Formed by INS‐1*: After the diabetic mice were generated, the INS‐1 cells were injected into the neo‐tissue niches of diabetic mice every third day for three times. Next, the injection site was replenished with TH every 6 d until day 27. During the process, the levels of blood glucose were monitored every third day following the first injection of INS‐1 with glucose meters (Roche, Basel, Switzerland). Monitoring continued until the end of experiment, when the mice were euthanized and tissues retrieved.

*IPGTT*: To detect the pancreas islet‐like xenograft tissue's responses to glucose challenge, IPGTT was conducted 27 d after the first injection of INS‐1 into ACG or THAG group. This assay could further assess the tissue's metabolic capacity in response to a glucose bolus. Briefly, animals were fasted overnight before receiving an intraperitoneal glucose bolus (2 g kg^−1^). The levels of blood glucose were monitored at 0, 15, 30, 60, 90, and 120 min after injection. Blood samples were also collected to measure the glucose‐stimulated secretion of rat insulin. In these experiments, blood was centrifuged for 10 min at 12 000 rpm and serum was stored at −80 °C until use.

*Assessment of Drug Metabolism Activity*: To analyze the drug metabolism activity, the humanized “liver‐like tissue” model formed by THLE‐3 cells in ACG or THAG group was challenged with debrisoquine, a reagent known to be metabolized differently by mice and humans. Sham‐operated C57/B6J mouse was used as control. Briefly, after debrisoquine (2 mg kg^−1^) was orally administrated, urine (0–8 h) was collected in acetate buffer (0.5 m, pH 5.0). KOH (1 n) was added to urine samples and incubated at 80 °C for 3 h, before being neutralized by equivalent volume of HCl (1 n). Acetonitrile containing 1% acetic acid was added and centrifuged (15 000 rpm, 4 °C, 5 min). The supernatant was subjected to liquid chromatography‐tandem mass spectrometry (LC/MS/MS). The area under the curve from time 0 until the last measurable urine concentration (AUC_0‐8_) was calculated using the linear trapezoidal rule. Metabolic ratios were determined by dividing AUC_0‐8_ of 4‐hyroxydebrisoqune by AUC_0‐8_ of debrisoquine.

*Histological Analyses*: The tissue samples were collected, frozen at optimal cutting temperature medium and cut into sections for H&E staining according to the manufacturer's instructions with slight modifications. The stained sections were photographed at different magnification times under a microscope. Under blindfold conditions under a standard light microscopy, the tissue was randomly examined. Meanwhile, frozen tissue sections for immunofluorescent staining were fixed with 4% paraformaldehyde and stained with primary antibody at 4 °C overnight. The primary antibodies included anti‐human CD31, anti‐mouse CD31, anti‐human histone, anti‐human RBP 2, anti‐human HNF 4 α, anti‐human Tuj1, anti‐human neuron (NeuN), anti‐human CYP 2D6, anti‐mouse SDF‐1, anti‐mouse Col I, anti‐mouse Col IV, anti‐mouse F4/80, anti‐mouse CD206, anti‐mouse E‐cadherin, anti‐mouse N‐cadherin, anti‐mouse smooth muscle actin, anti‐mouse vimentin, anti‐rat insulin, and anti‐rat Nkx6.1. Next, the sections were incubated with secondary antibody Alexa Fluor (Life Technologies) for 1 h at room temperature, followed by 4,6‐diamidino‐2‐phenylindole (DAPI) for nuclear staining. All fluorescence including GFP bioluminescence were captured with a Nikon confocal microscope (C2+, Nikon, Tokyo, Japan) and analyzed using Nis‐element advanced research software (Nikon).

*Statistical Analysis*: The results are expressed as mean ± standard deviation (SD). Data were statistically analyzed using Prism software (GraphPad) and assessed for normality or homogeneity of variance with D‐test and Levene test. Differences between multiple groups were compared using one‐way analysis of variance (ANOVA) with Dunnett's tests or, if appropriate, repeated measures ANOVA test with post hoc Bonferroni correction. Differences between two groups were evaluated using the unpaired Student's *t*‐test. A value of *P* < 0.05 was considered significant and “ns” stands for “not significant.”

## Conflict of Interest

The authors declare no conflict of interest.

## Supporting information

Supplementary
