# Supplementary material for: Engineering a Tumor Microenvironment‐Mimetic Niche for Tissue Regeneration with Xenogeneic Cancer Cells
Source: Adv Sci (Weinh). 2018 Jan 2;5(3):1700666. doi: 10.1002/advs.201700666 (PMC5867037; doi:10.1002/advs.201700666)
Supplement: Supplementary file 1 — Supplementary [file ADVS-5-1700666-s001.pdf]

Copyright WILEY-VCH Verlag GmbH & Co. KGaA, 69469 Weinheim, Germany, 2017.

ADVANCED SCIENCE
Open Access

Supporting Information

for *Adv. Sci.,* DOI: 10.1002/advs.201700666

Engineering a Tumor Microenvironment-Mimetic Niche for Tissue Regeneration with Xenogeneic Cancer Cells

Zhenzhen Wang, Chunming Wang, Ayipaxia Abudukeremu, Xiaying Rui, Shang Liu, Xiaoyi Zhang, Min Zhang, Junfeng Zhang,* and Lei Dong**

Copyright WILEY-VCH Verlag GmbH & Co. KGaA, 69469 Weinheim, Germany, 2016.

Supplementary Information

Engineering a Tumour Microenvironment-mimetic Niche for Tissue Regeneration with Xenogeneic Cancer Cells

Zhenzhen Wang[1], Chunming Wang[2,], Ayipaxia Abudukeremu[1], Xiaying Rui[1], Shang Liu[1], Xiaoyi Zhang[3], Min Zhang[1], Junfeng Zhang[1,*], Lei Dong[1,*]*

1. State Key Laboratory of Pharmaceutical Biotechnology, School of Life Sciences, Nanjing University, 163 Xianlin Avenue, Nanjing 210093, China.
2. State Key Laboratory of Quality Research in Chinese Medicine, Institute of Chinese Medical Sciences, University of Macau, Taipa, Macau SAR.
3. Department of Chemistry, Emory University, 1515 Dickey Drive, Atlanta, GA 30322, USA

* Corresponding Authors: L.D., Email: leidong@nju.edu.cn; C.M.W., Email: cmwang@umac.mo; J.F.Z., Email: Jfzhang@nju.edu.cn.

Table S1. Pathways Significantly Enriched by the TH Proteins Related to Immune Response, Metabolic Activity or Other Signal Transduction (By Gene Ontology Analysis).

Items	Related Pathway
**Immune Response**	T cell receptor signalling pathway
	Natural killer cell mediated cytotoxicity
	Leukocyte transendothelial migration
	Fc gamma R-mediated phagocytosis
	Fc epsilon RI signalling pathway
	Complement and coagulation cascades
	Chemokine signalling pathway
	B cell receptor signalling pathway
	Antigen processing and presentation
**Metabolic Activity**	Purine metabolism
	Glycolysis / Gluconeogenesis
	Galactose metabolism
	Fructose and mannose metabolism
	Carbon metabolism
	Biosynthesis of antibiotics
	Amino sugar and nucleotide sugar
	Glutathione metabolism
**Signal Transduction and Interaction**	Sphingolipid signaling pathway
	Ras signalling pathway
	Rap1 signalling pathway
	MAPK signalling pathway
	ErbB signalling pathway
	cGMP-PKG signalling pathway
	VEGF signalling pathway

Table S2. List of Soluble Factors Detected by Mouse Cytokine Array kit and Mouse Angiogenesis Array Kit.

Category (Number[a])	Related Protein
Growth factor (14)	EGF; VEGF; FGF acidic; PDGF-AB; PD-ECGF; HB-EGF; HGF; FGF basic; KGF; NOV; PlGF-2; IGFBP-1; IGFBP-2; IGFBP-3
Coagulation factor (5)	Thrombospondin-2; Serpin E1; Platelet Factor 4; ADAMTS1; Coagulation Factor III
Extracellular Matrix (5)	MMP-8 (pro form); TIMP-4; TIMP-1; MMP-9 (pro and active form); MMP-3 (pro and mature form);
Angiogenesis (13)	Endothelin-1; Endoglin; DLL4; Leptin; Angiopoietin-3; Pentraxin-3; VEGF-B; Angiopoietin-1; PDGF-AA; Angiogenin; Cyr61; SerpinF1; Endostatin
Chemokines (19)	CXCL16; SDF-1; Fractalkine; IP-10; MCP-1; sICAM-1; RANTES; MIG; IL-3; MCP-5; MIP-2; BLC; Eotaxin; I-TAC; I-309; JE; TARC; MIP-1β; MIP-1α
Pro-inflammatory factor (15)	C5/C5a; IL-6; IL-1α; TREM-1; IL-1β; IL-23; IL-5; IL-17; IL-1ra; IL-12 p70; IFN-γ; IL-2; IL-27 ;TNF-α; IL-7
Anti-inflammatory factor (4)	IL-10; IL-16; IL-13; IL-4
Colony-stimulating factor (3)	M-CSF; G-CSF; GM-CSF

a. The total number of related proteins in each category.

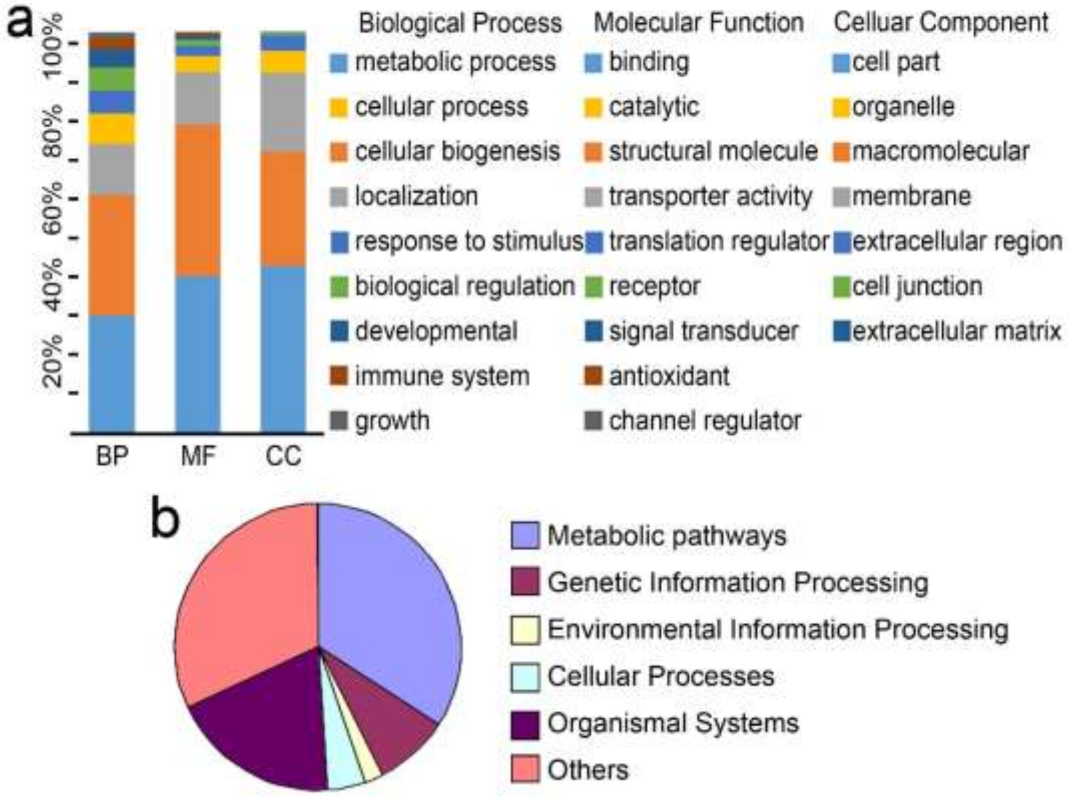

Figure S1. Proteomics analysis of TH from the soluble extract of murine sarcoma. a. Hierarchical cluster analysis of TH proteins in LC-MS profile according to independent ontology; b. Analysis of pathways orchestrated by TH proteins performed by Gene Ontology Pathway.

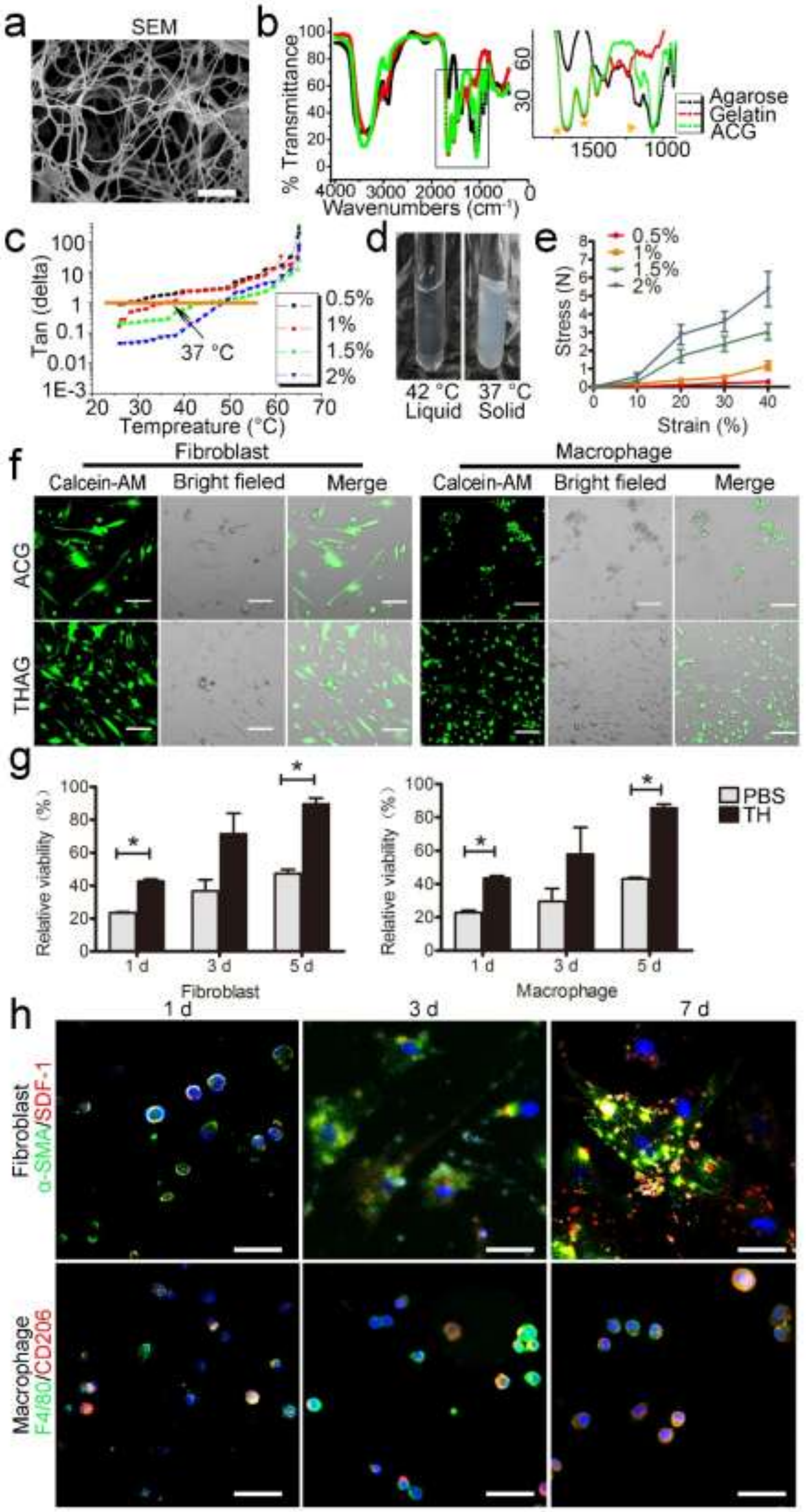

Figure S2. ACG promotes cell adhesion and growth. a. SEM image of a

fractured, lyophilised hydrogel prepared from a 1% ACG solution, revealing a highly porous and interconnected interior structure with an average pore size of approximately 10-20 μm (Scale bar=10 μm); b. Fourier transform infrared spectroscopy (FTIR) spectra of agarose, gelatin and ACG. The asterisks at 1655 cm^{-1} and 1541 cm^{-1} indicated the C=O peak from, the COOH group; the triangle at 1094 cm^{-1} represented hydroxyl groups of agarose; c. Rheological property of ACG hydrogel at different concentrations. Representation of tan delta as a function of temperature in a multifrequency oscillation experiment for hydrogel aqueous solutions of different concentrations. The temperature at which tan delta 1 was considered as the gelation temperature and the arrow indicates 1% ACG hydrogel's gelation temperature is about 37 ^{o}C; d. The liquefied 1% THAG formed into THAG gel as the temperature decreased from 42 ^{o}C to 37 ^{o}C or lower; e. The stress-strain profiles of ACG hydrogel at different concentration until rupture of the specimens are shown; f. Morphology of fibroblasts and macrophages on the THAG or ACG gel at 37 ^{o}C (bright field) with the live cells stained by Calcein-AM (green) (Scale bar=125 μm); g. CCK-8 test of fibroblasts and macrophages on the gel treated as in

panel f at indicated time, with monolayer cultured cells on the dish as control. h. Representative immunostaining photographs of fibroblasts (SDF-1, red; α-SMA, green) and macrophages (CD206, red; F4/80, green) encapsulated in the THAG (Scale bar=50 μm). Images are representative of three independent experiments. Results are shown as mean ± SD. * $p<0.05$ after ANOVA with Dunnett's tests.

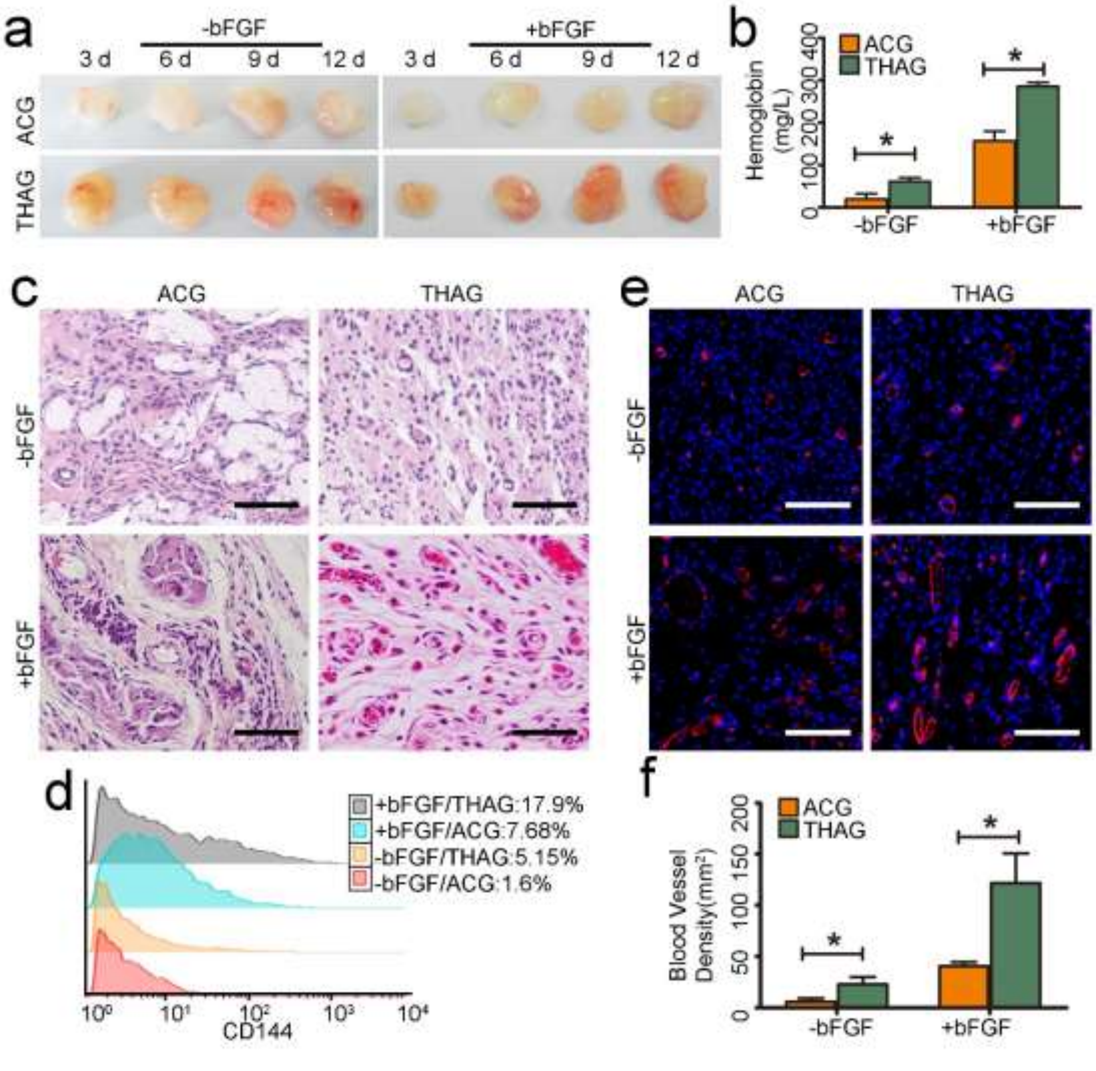

Figure S3. Basic fibroblast growth factor (bFGF) enhances angiogenesis. a. The

photograph of neo-tissue niches formed in THAG or ACG group with or without basic fibroblast growth factor at indicated time; b. The haemoglobin content in neo-tissue 9 days after the first injection in each group treated as in panel a (n=7-9 per group); c. Representative images of H&E staining for the neo-tissue niches treated as in panel a (Scale bar=100 μm); d. FACS analysis of CD144 (marker of endothelial cells) in neo-tissue niches in each group treated as in panel a; e. Representative immunostaining photographs of CD31. f. Blood vessel density calculated by the confocal images shown as in e. Images are representative of three independent experiments. Results are shown as mean ± SD. *p<0.05 after ANOVA with Dunnett's tests.

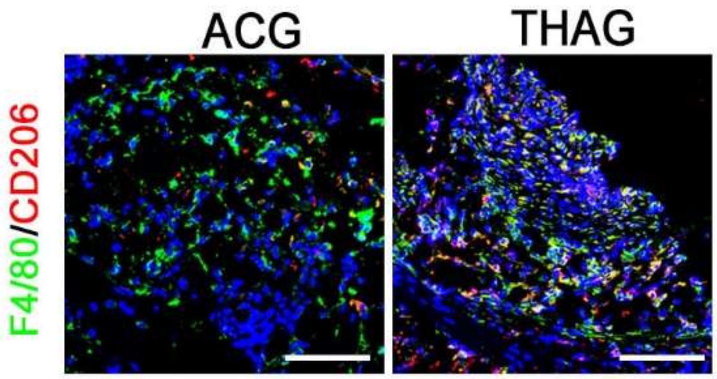

Figure S4. Representative immunostaining photographs of CD206 (type 2 macrophage marker) and F4/80 in neo-tissues implanted with THAG or ACG

(Scale bar=100 μm).

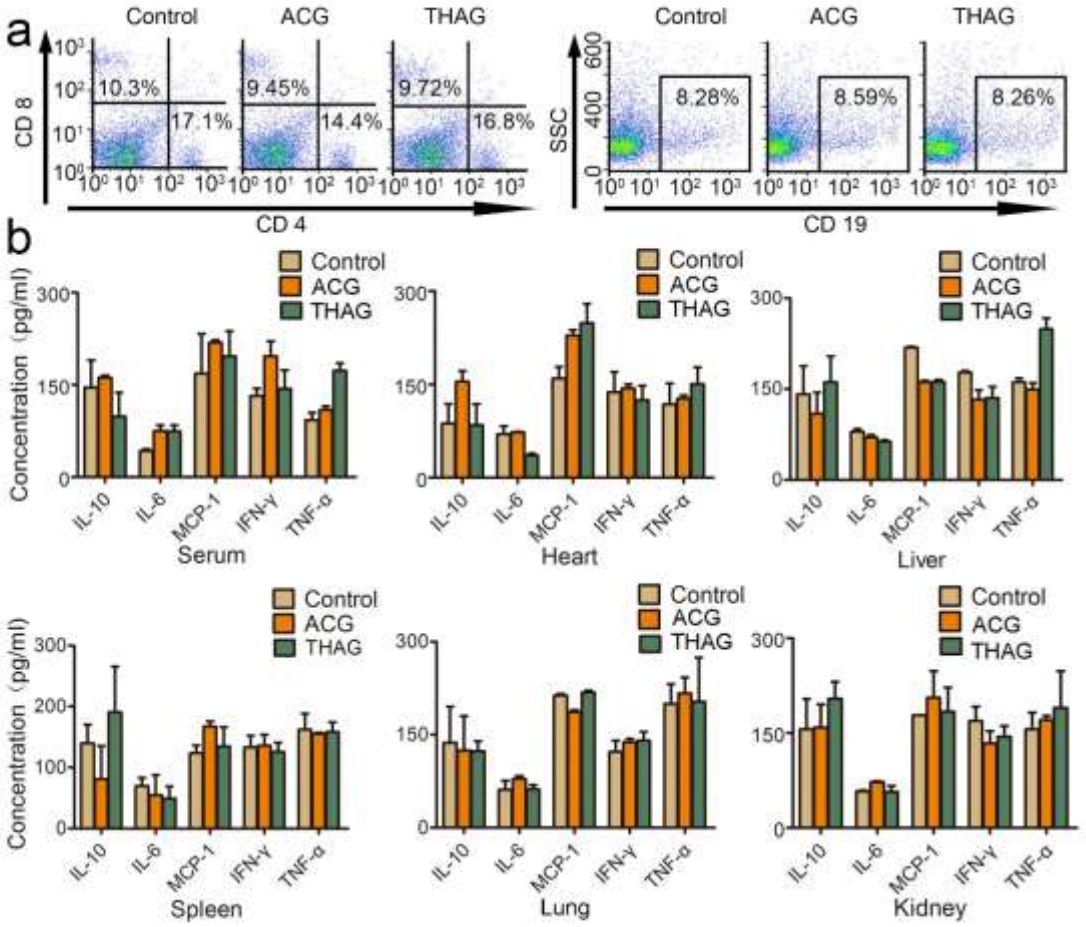

Figure S5. *In vivo* response to THAG or ACG implantation. a. The proportion of T and B cells and representative flow cytometry analysis in the peripheral blood 9 days after the first injection of THAG or ACG, with sham-operated mice serving as control; b. The levels of inflammatory factors (IL-10, IL-6, MCP-1, IFN-γ, TNF-α and IL-12p70) in the serum and various tissues (heart, liver, spleen, lung and kidney) of three groups treated as in panel a measured by ELISA. Results are shown as mean

± SD (n = 7 mice per group).

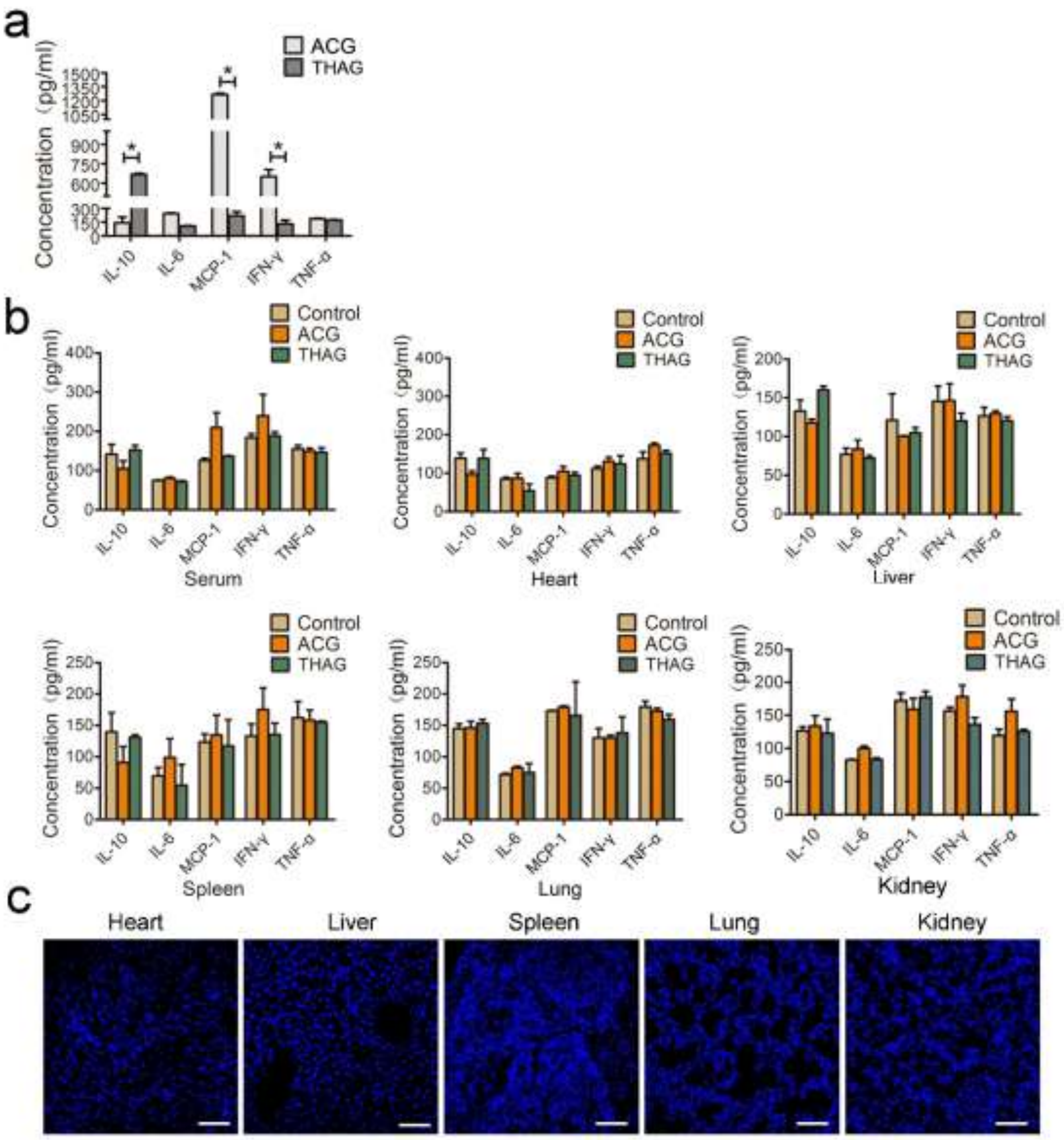

Figure S6. ***In vivo*** **response to the implantation of trans-species GFP-293T cells lines-laden THAG.** a. The levels of inflammatory factors (IL-10, IL-6, MCP-1, IFN-γ, TNF-α and IL-12p70) in the neo-tissue site 9 days after the first injection of trans-species GFP-293T cells in THAG or ACG (n = 7 mice per group); b. The level

of inflammatory factors (IL-10, IL-6, MCP-1, IFN-γ, TNF-α and IL-12p70) in the serum and various tissues (heart, liver, spleen, lung and kidney) in GFP-293T cells lines-laden THAG or ACG group, , with sham-operated mice serving as control (n = 7 mice per group); c. Representative images from confocal laser scanning microscopy observation of human histone in other tissues 9 days after the first injection of GFP-293T in THAG (Scale bar=100 μm). Images are representative of three independent experiments. Results are shown as mean ± SD. *p<0.05 after ANOVA with Dunnett’s tests.

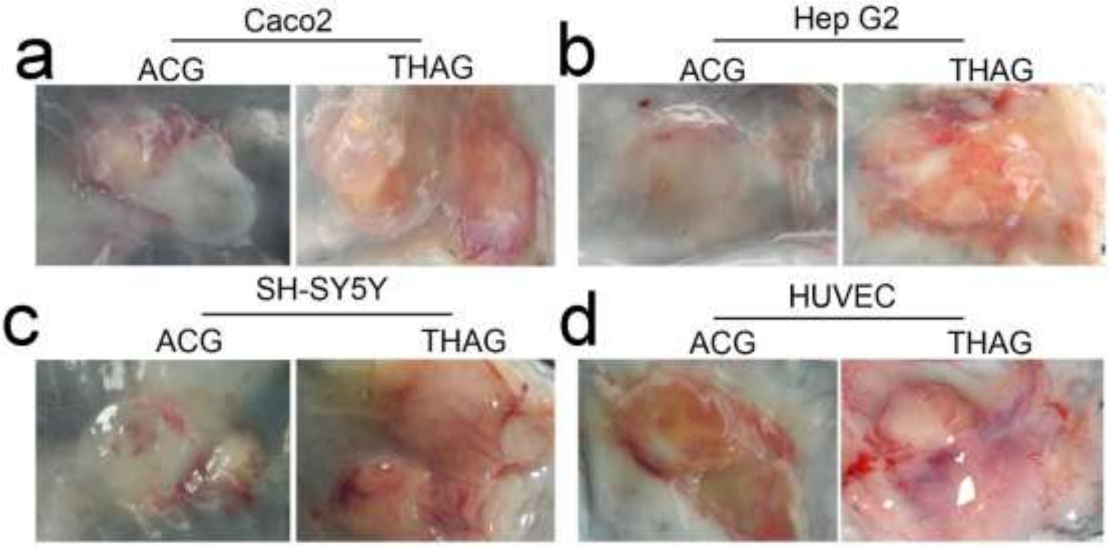

Figure S7. The representative photograph of Caco2 (a), Hep G2 (b), SH-SY5Y (c) and HUVEC (d) cells-laden THAG or ACG 9 days after first implantation of the

human cells.

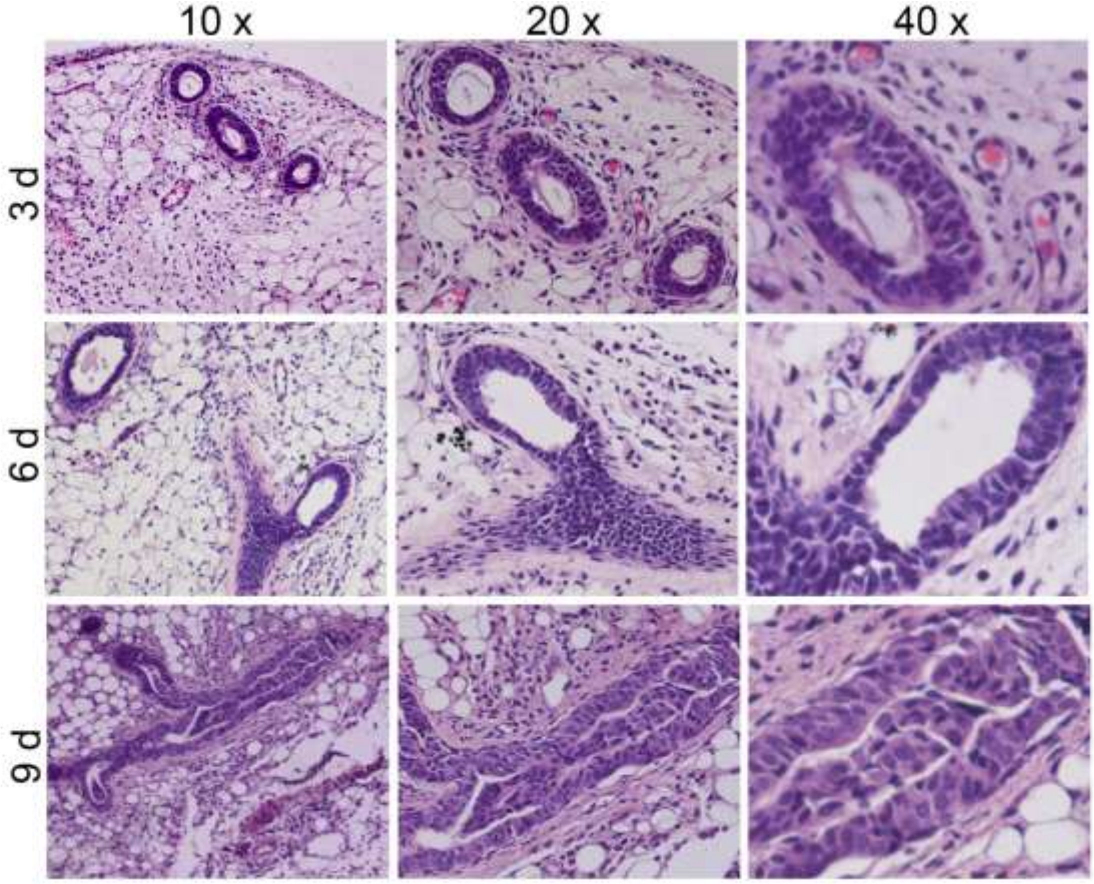

Figure S8. Histological photograph of the epithelioid structure with different magnification in Caco2 cells injection site at indicated time in THAG group with H&E staining.

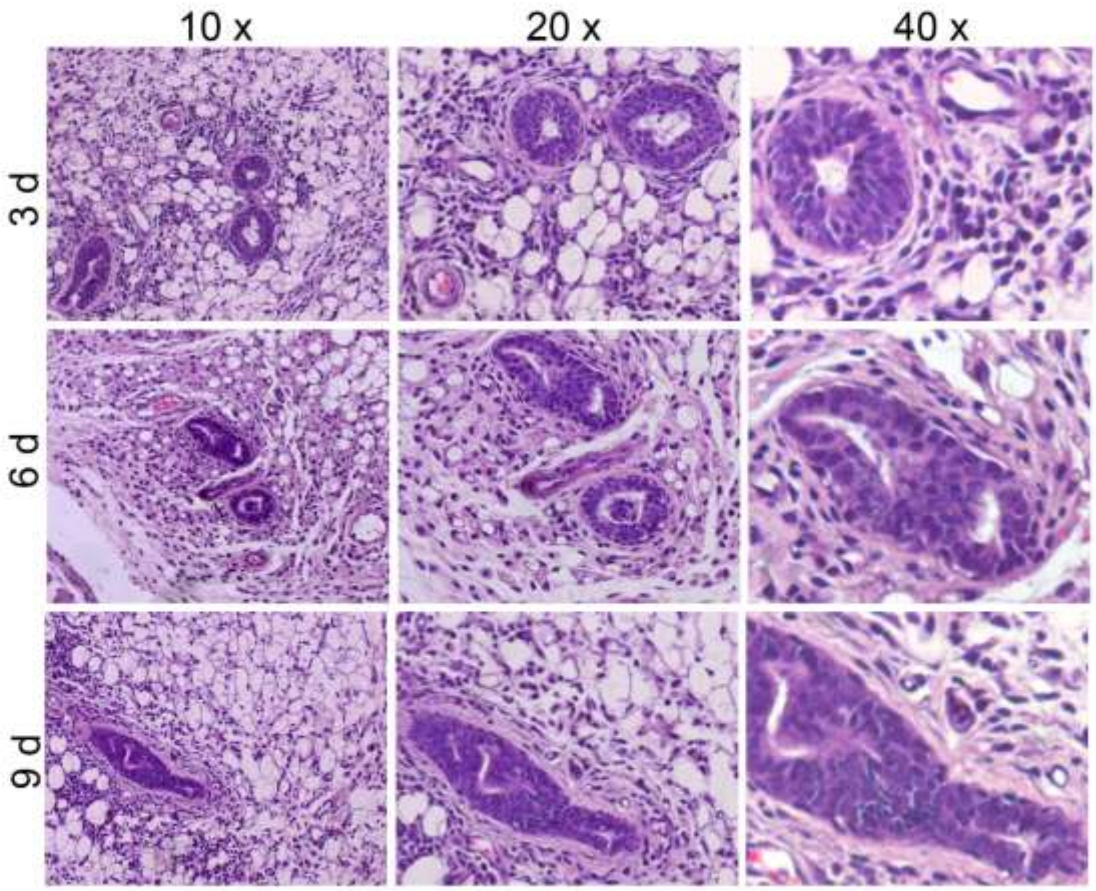

Figure S9. Histological examination (H&E staining) of the epithelioid structure with various magnifications in Hep G2 cells-laden THAG at indicated time.

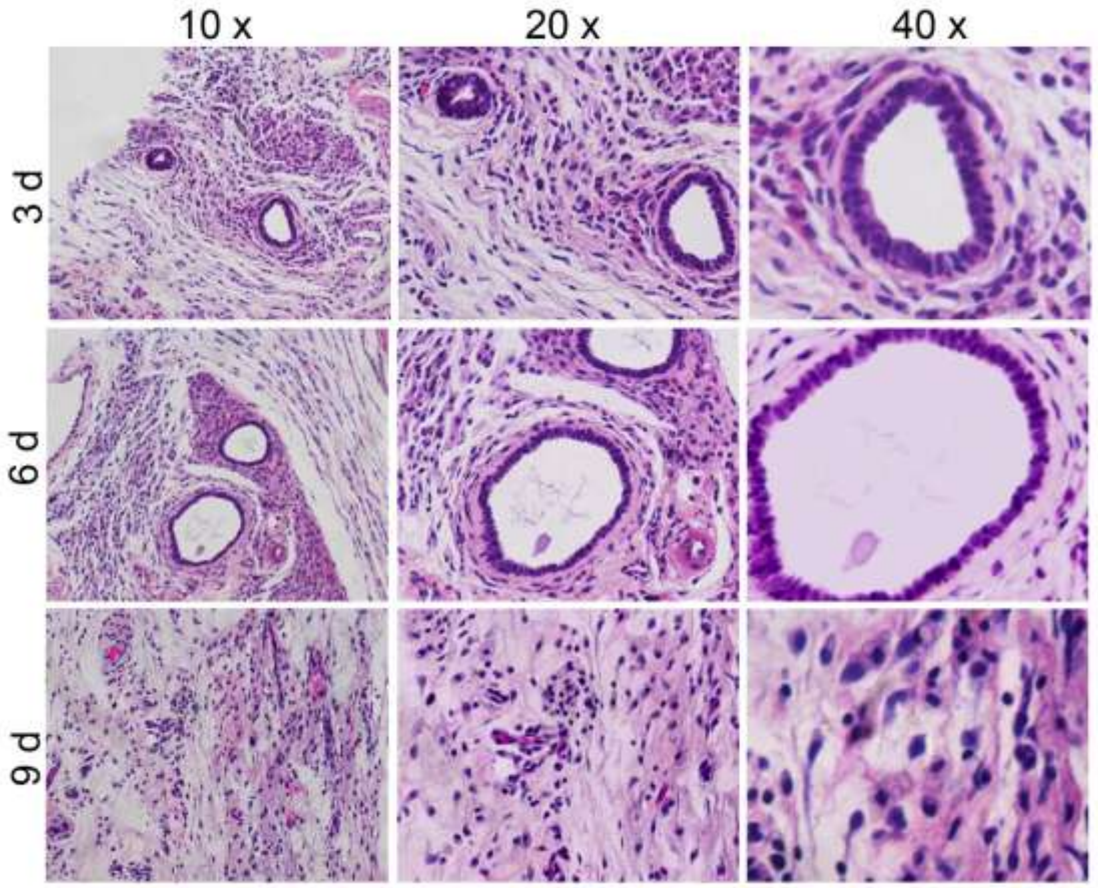

Figure S10. Histological examination (H&E staining) of neuroepithelial cysts, the early stage structure of neural differentiation, and extended dendrites of a neural network with different magnification in SH-SY5Y cells-laden THAG at indicated time.

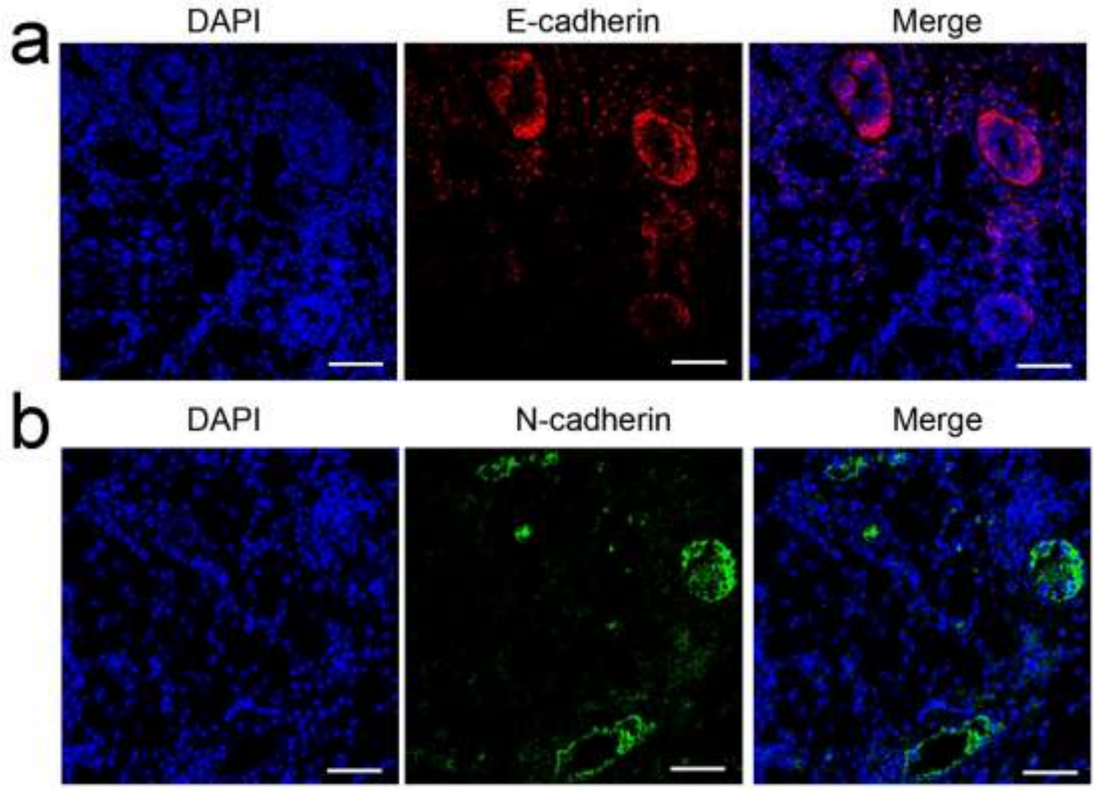

Figure S11. a. Representative immunofluorescent staining of E-cadherin 3 days after the first injection of SH-SY5Y cells (Scale bar=100 μm); b. Representative immunofluorescent staining of N-cadherin in the SH-SY5Y cell injection site at day 8 (5 days post the second treatment) (Scale bar=100 μm).

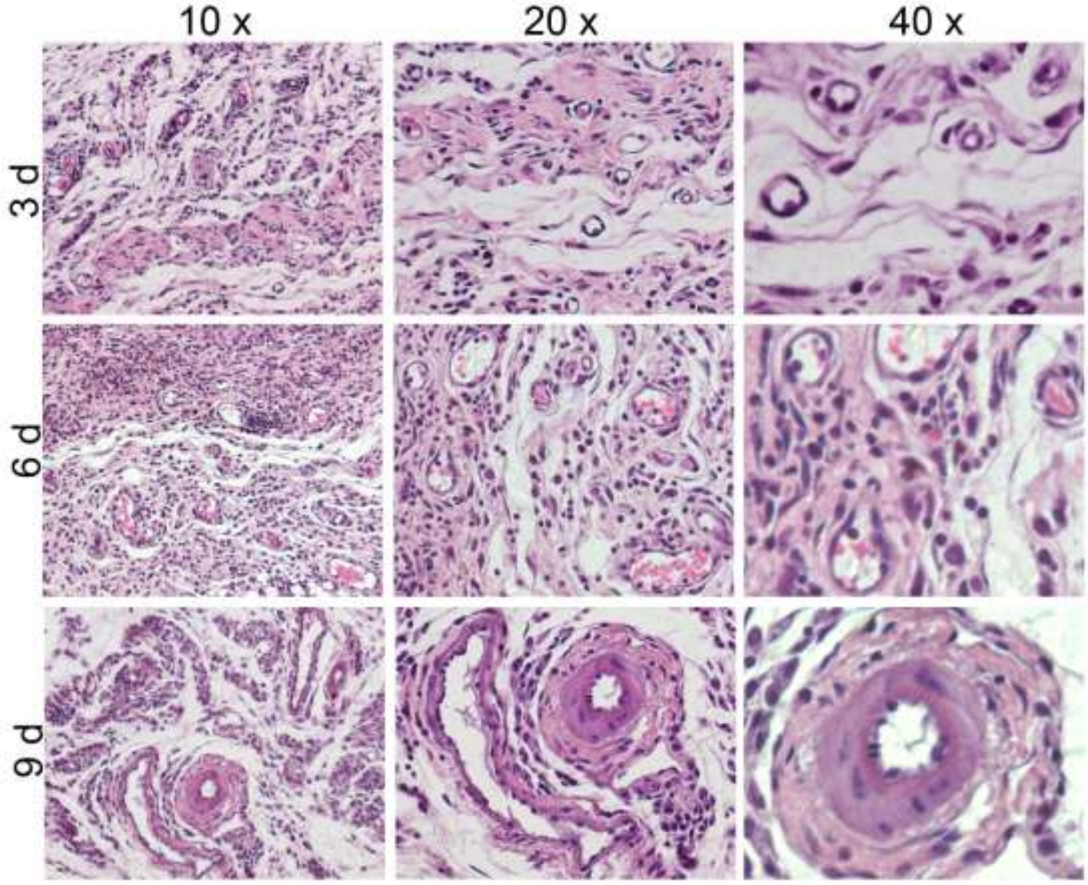

Figure S12. Histological examination (H&E staining) of vascular structures with various magnifications in HUVEC cells-laden THAG at indicated time.

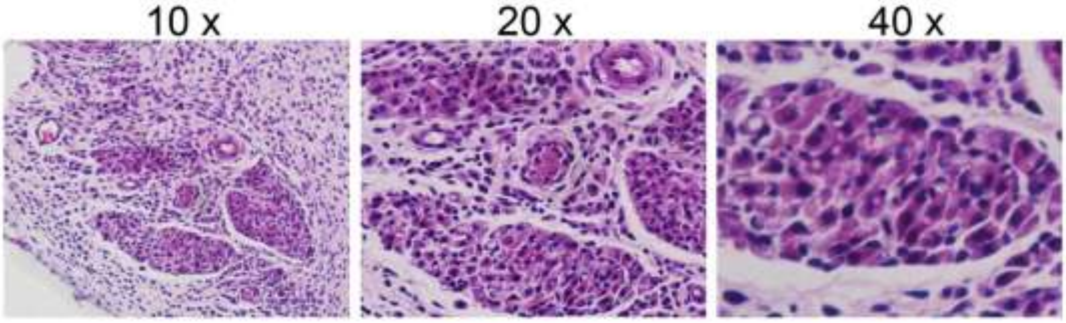

Figure S13. Histological examination (H&E staining) of Langerhans-like structures with various magnifications in INS-1-laden THAG 27 days after the first injection.

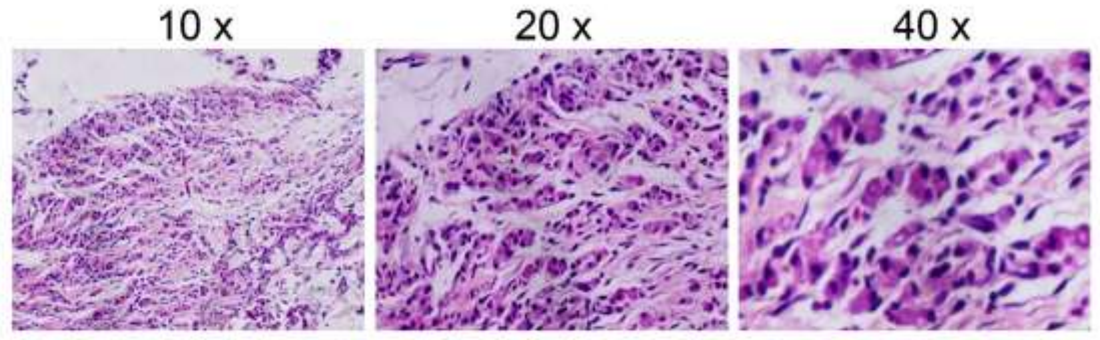

Figure S14. Representative histological regions with different magnification showing the larger polygonal cells with the analogous typical morphology of liver cells 9 days after the first injection.
